# Tropism of SARS-CoV-2 for human cortical astrocytes

**DOI:** 10.1073/pnas.2122236119

**Published:** 2022-07-12

**Authors:** Madeline G. Andrews, Tanzila Mukhtar, Ugomma C. Eze, Camille R. Simoneau, Jayden Ross, Neelroop Parikshak, Shaohui Wang, Li Zhou, Mark Koontz, Dmitry Velmeshev, Clara-Vita Siebert, Kaila M. Gemenes, Takako Tabata, Yonatan Perez, Li Wang, Mohammed A. Mostajo-Radji, Martina de Majo, Kevin C. Donohue, David Shin, Jahan Salma, Alex A. Pollen, Tomasz J. Nowakowski, Erik Ullian, G. Renuka Kumar, Ethan A. Winkler, Elizabeth E. Crouch, Melanie Ott, Arnold R. Kriegstein

**Affiliations:** ^a^Department of Neurology, University of California, San Francisco, CA 94143;; ^b^The Eli and Edythe Broad Center of Regeneration Medicine and Stem Cell Research, University of California, San Francisco, CA 94143;; ^c^School of Biological and Health Systems Engineering, Arizona State University, Tempe, AZ 85281;; ^d^Gladstone Institutes, San Francisco, CA 94158;; ^e^Department of Medicine, University of California, San Francisco, CA 94143;; ^f^University of California, San Francisco Biomedical Sciences Graduate Program, San Francisco, CA 94143;; ^g^Department of Anatomy, University of California, San Francisco, CA 94143;; ^h^Department of Ophthalmology, University of California, San Francisco, CA 94143;; ^i^Center for Regenerative Medicine and Stem Cell Research, The Aga Khan University, Karachi, 74800, Pakistan;; ^j^Department of Neurological Surgery, University of California, San Francisco, CA 94143;; ^k^Department of Pediatrics, University of California, San Francisco, CA 94143

**Keywords:** astrocyte reactivity, organoid models, SARS-CoV-2 tropism

## Abstract

The severe acute respiratory syndrome coronavirus 2 (SARS-CoV-2) readily infects a variety of cell types impacting the function of vital organ systems, with particularly severe impact on respiratory function. Neurological symptoms, which range in severity, accompany as many as one-third of COVID-19 cases, indicating a potential vulnerability of neural cell types. To assess whether human cortical cells can be directly infected by SARS-CoV-2, we utilized stem-cell-derived cortical organoids as well as primary human cortical tissue, both from developmental and adult stages. We find significant and predominant infection in cortical astrocytes in both primary tissue and organoid cultures, with minimal infection of other cortical populations. Infected and bystander astrocytes have a corresponding increase in inflammatory gene expression, reactivity characteristics, increased cytokine and growth factor signaling, and cellular stress. Although human cortical cells, particularly astrocytes, have no observable ACE2 expression, we find high levels of coronavirus coreceptors in infected astrocytes, including CD147 and DPP4. Decreasing coreceptor abundance and activity reduces overall infection rate, and increasing expression is sufficient to promote infection. Thus, we find tropism of SARS-CoV-2 for human astrocytes resulting in inflammatory gliosis-type injury that is dependent on coronavirus coreceptors.

## Significance

Neurological and neuropsychiatric symptoms occur in as many as one-third of patients with or recovering from COVID-19. To address severe acute respiratory syndrome coronavirus 2 (SARS-CoV-2) neural tropism, we evaluated the vulnerability of primary developing and adult cortical tissue and stem-cell-derived cortical organoids to SARS-CoV-2 infection. We observe robust infection and viral replication in human cortical astrocytes; they become reactive, have increased growth factor signaling, and activate cellular stress. Despite infection in astroglial cells, there is minimal infection of other neural cell types, including neurons. Surprisingly, we discover that astrocyte infection is unlikely to be mediated by the predominant coronavirus receptor, ACE2, and instead observe alternative glycoproteins, CD147 and DPP4, expressed on astrocytes; they are necessary and sufficient for SARS-CoV-2 infection.

The severe acute respiratory syndrome coronavirus 2 (SARS-CoV-2) causes the life-threatening illness COVID-19 and is responsible for a global pandemic resulting in more than 5.8 million deaths worldwide. Although SARS-CoV-2 infection can cause catastrophic, life-threatening damage to respiratory function, the capacity to infect other cell types and disrupt function of additional organ systems is a subject of intense study. Strikingly, many patients suffering with or having recovered from COVID-19 present with a range of neurological symptoms including seizures; encephalopathy; stroke; headaches; dizziness; short-term memory loss; loss of smell and taste; confusion; a general inability to focus; and new or recurring neuropsychiatric symptoms, like anxiety and depression (1–5). As many as one-third of individuals, 6 mo after recovering from COVID-19 infection, are diagnosed with neurological or neuropsychiatric conditions (6). Additionally, individuals with mental health diagnoses are more susceptible to coronavirus infection and have impaired long-term health outcomes (7).

It is unclear whether the range of neurological symptoms are a result of direct infection of the neural tissue or a secondary consequence of widespread inflammation downstream of viral infection in other tissues. Several studies have demonstrated how inflammation contributes to systemic problems in a variety of organ systems, including the central nervous system (CNS) (8, 9). However, SARS-CoV-2 can infect neurons in the nasal epithelium, a potential mode of entry to the CNS from the periphery, and the presence of viral RNA has been detected in neural tissues in patients (10). Recent studies report mixed findings regarding the presence of coronavirus viral RNA and antibodies in the cerebral spinal fluid (CSF) of COVID-19 patients (3, 8, 11, 12). However, choroid plexus organoids containing the cell type that produces CSF can be readily infected by SARS-CoV-2 *in vitro* (13, 14), suggesting possible viral access to CSF. Additionally, the virus can infect and disrupt brain vasculature. Studies of postmortem brain tissue from severely infected COVID-19 patients have reported widespread inflammation in the brainstem, choroid plexus, and brain parenchyma characterized by infiltration of immune cells including microglia and T cells, as well as infection of cranial nerves, microvascular injury, fibrinogen leakage, and extensive astrogliosis (8, 15, 16). Together, these studies suggest the capacity for viral transmission into the CNS through leaky vasculature, the nasal epithelium, and/or CSF.

The vulnerability of particular cell types in the brain and the impact on neurological health and function require in-depth study, and human stem-cell-derived neural models have been utilized to evaluate viral tropism (17, 18). Studies of cerebral organoids, which are reflective of developmental stages, suggest that *in vitro* neurons may be vulnerable to SARS-CoV-2 infection. However, reports regarding susceptibility of neurons from different brain regions have been mixed across organoid studies (13, 14, 19). Recently, studies have begun to explore the vulnerability of nonneuronal populations, including vascular pericytes and glial cells, using stem-cell-derived organoid and assembloid models (20, 21). Additionally, studies exploring the loss of smell identified viral tropism of nonneural support cells and vascular cells in the olfactory epithelium (22). Here, we utilized primary cortical tissue from both the developing and the adult brain, paired with cortical organoid models across neurogenic and gliogenic stages, to evaluate which human neural cell types can be directly infected by SARS-CoV-2 and at what stages of maturation. In primary cortical tissue cultures and cortical organoids exposed to SARS-CoV-2, we observed significant infection and viral replication in immature and mature astrocytes but minimal infection in other neural cell types. As a response to infection, we observed widespread inflammation, cytokine secretion, and reactivity in astrocytes. However, cortical astrocytes do not express observable levels of ACE2, the canonical SARS-CoV-2 receptor, suggesting that the virus may use another means of entry. We observed that SARS-CoV-2 cofactors CD147 and DPP4 are highly expressed in infected astrocytes. Reducing the abundance of CD147 or the activity of DPP4 reduced infection, whereas increasing expression of these receptors promoted infection, suggesting a role in viral entry or propagation. Our study provides evidence of SARS-CoV-2 tropism for human astrocytes with implications for the cellular vulnerability of the human brain and downstream consequences to neurological function.

## Results

### SARS-CoV-2 Infects Astrocytes in Primary Developing Human Cortical Tissue.

**Fig. 1.**
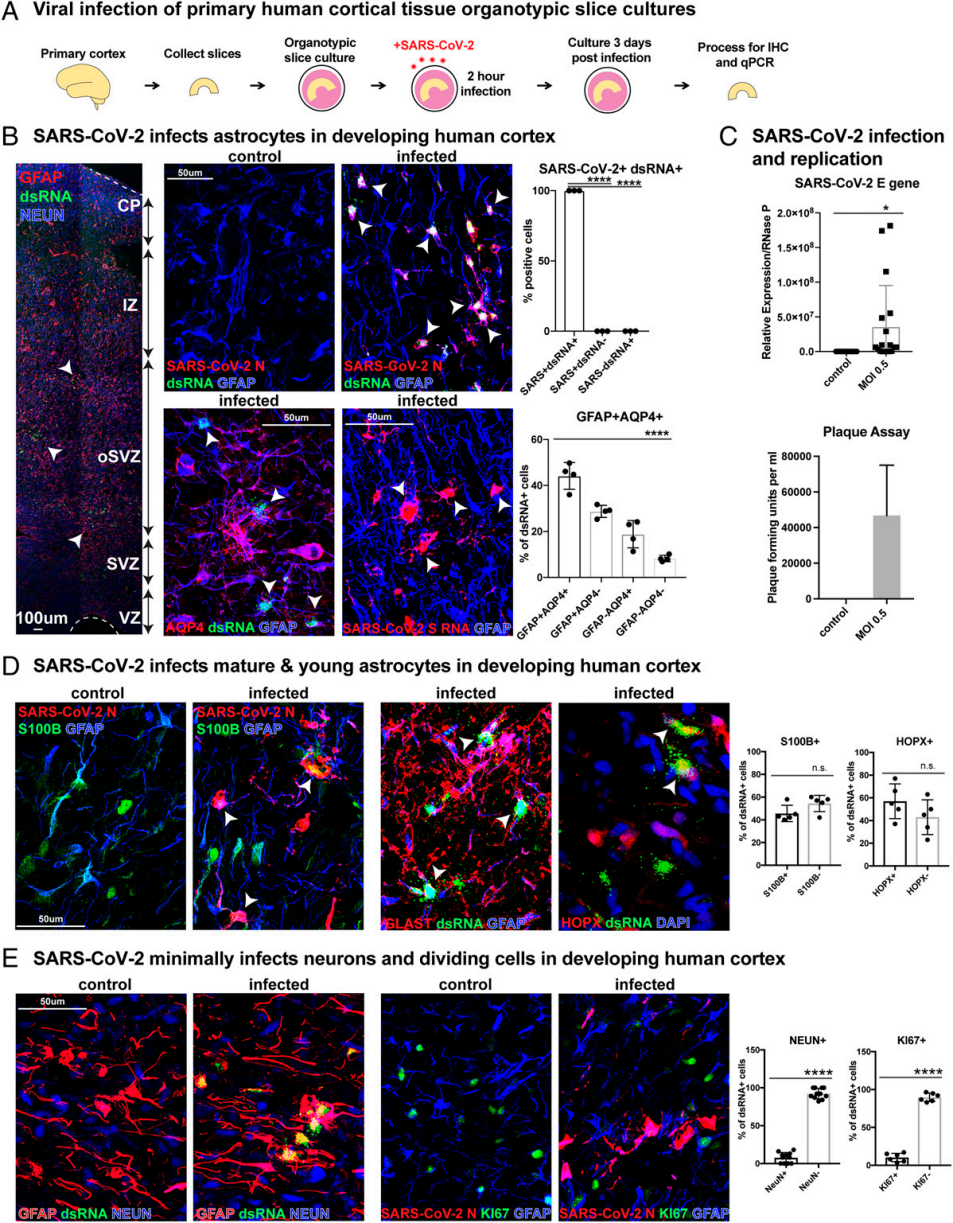
SARS-CoV-2 infects astrocytes in developing human cortex. (*A*) Experimental paradigm for viral infection of human cortical tissue from GW 19 to 23. Cortical tissue was sliced, exposed to SARS-CoV-2 for 2 h, and cultured for 72 h. Samples were processed for immunostaining and qPCR. (*B*) SARS-CoV-2 infects GFAP+AQP4+ astrocytes in developing human cortex. All (100%) infected cells coexpress SARS-CoV-2+ nucleocapsid (N) and dsRNA, indicating viral infection and replication (one-way ANOVA, SARS-CoV-2+dsRNA+ vs. SARS-CoV-2-dsRNA+ ****P < 0.0001, SARS-CoV-2+dsRNA+ vs. SARS-CoV-2+dsRNA− ****P < 0.0001, error bars represent SD, *n* = 3 technical replicates from two biological samples). More than 90% of infected cells coexpress markers of astrocytes and SARS-CoV-2 infection (white arrowheads; one-way ANOVA: GFAP+AQP4+ vs. GFAP-AQP4− ****P < 0.0001, error bars represent SD, *n* = 4 technical replicates from three biological samples from stages GW 19, 22, 23). (*C*) Plaque assay was used to identify SARS-CoV-2 propagation in astrocytes. Astrocytes were exposed to the virus for 2 h and washed, and the media were changed. Supernatant from infected astrocytes was then cultured on Vero cells. Vero cells were fixed and plaque forming units quantified, where none were observed in control but >40,000 observed in cells treated with MOI 0.5 supernatant (*n* = 2, error bars represent SD). SARS-CoV-2 viral RNA can also be detected in slice cultures exposed to MOI 0.5 by qRT-PCR (unpaired Student’s *t* test, *P < 0.0149, *n* = 3 slices/condition from two biological replicates across three independent qRT-PCRs). (*D*) Mature S100B+ or GLAST+ and immature HOPX+ astrocytes demonstrate viral tropism, as approximately half of infected cells express these markers as indicated by white arrowheads (unpaired Student’s *t* test, S100B: *P* = 0.088 HOPX: *P* = 0.186, error bars represent SD, *n* = 3 biological samples from stages GW 19, 22, 23 from five technical replicates). (*E*) SARS-CoV-2 minimally infects neurons and dividing cells. Few infected dsRNA+ SARS-CoV-2+ cells are NEUN+ cortical neurons or KI67+ dividing cells, as fewer than 8% are NEUN+, and fewer than 11% are KI67+ (unpaired Student’s *t* test: NEUN ****P < 0.0001, KI67 ***P < 0.0001, error bars represent SD; *n* = 3 biological samples from six technical replicates).

**Fig. 2.**
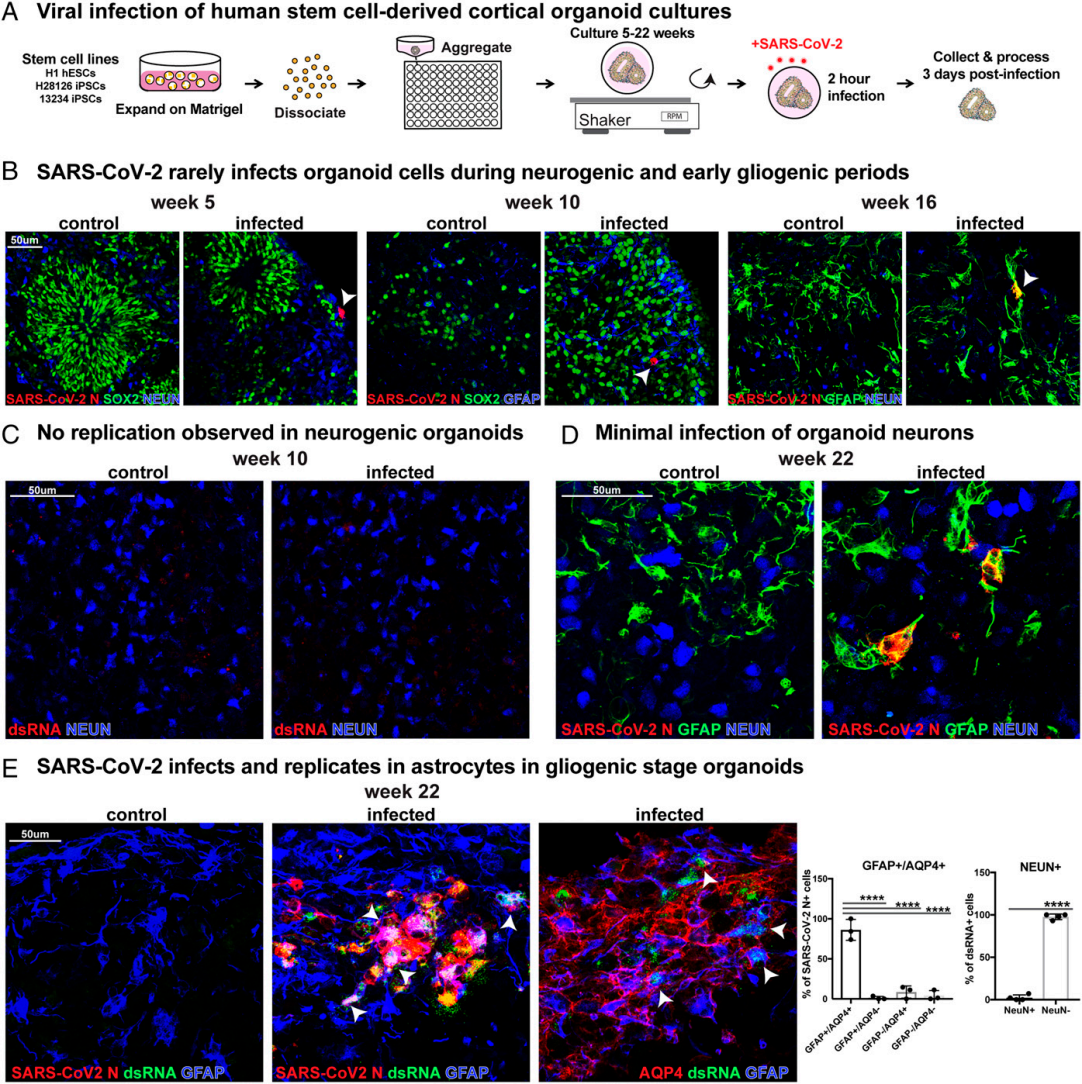
SARS-CoV-2 infects astrocytes in cortical organoids. (*A*) Experimental paradigm for viral infection of induced pluriptotent stem cell (iPSC)-derived cortical organoids. Organoids from differentiation weeks 5, 10, 16, and 22 were infected with SARS-CoV-2. (*B*) Cells in organoids are only rarely infected at 5, 10, or 16 wk, as indicated by SARS-CoV-2 N expression (white arrowheads). These differentiation timepoints correspond to neurogenic and early gliogenic periods. (*C*) Although rare cells are infected and coexpress SARS-CoV-2 at neurogenic stages, there is no observed dsRNA+ viral replication at these timepoints. (*D*) In week 22 organoids, which correspond to the gliogenic period of differentiation, infection is readily observed. GFAP+ astrocytes coexpress SARS-CoV-2 N, but NEUN+ neurons are very rarely infected. (*E*) SARS-CoV-2 preferentially infects and replicates in astrocytes in gliogenic stage organoids. In total, 96% of infected cells stain positive for astrocyte markers (GFAP or AQP4), while only 2% express a neuronal marker (NEUN+). White arrowheads indicate SARS-CoV-2+ dsRNA+ GFAP+ AQP4+ astrocytes (one-way ANOVA: GFAP+AQP4+ vs. GFAP+AQP4−/GFAP−AQP4+/GFAP−AQP4− ****P < 0.0001, GFAP+AQP4+; NEUN: unpaired Student's *t* test: ****P < 0.00001, error bars represent SD, *n* = 4 organoids from two stem cell lines).

To evaluate the capacity of SARS-CoV-2 to directly infect human cortical cells, we exposed organotypic slice cultures of developing human cortical samples from gestational weeks (GW) 19 to 23 to SARS-CoV-2 for 2 h and evaluated the slice cultures after 72 h (Fig. 1*A*). Infected cells were identified using a coronavirus nucleocapsid (N) antibody or SARS-CoV-2 Spike (S) RNA probe, and viral replication was documented by the presence of double-stranded RNA (dsRNA). To evaluate the vulnerability of neural cell types for infection, we utilized markers for cortical progenitors, neurons, and glial cells, including astrocytes and oligodendrocytes. Strikingly, we observed abundant infection in astrocytes, as indicated by the presence of viral N protein, S RNA, and dsRNA, with 90% of infected cells expressing glial fibrillary acidic protein (GFAP) or Aquaporin 4 (AQP4) throughout the outer subventricular zone (SVZ) of primary cortical tissue (Fig. 1*B*). We found colocalization of viral N protein and dsRNA in 100% of infected astrocytes, indicating both efficient infection and viral replication in these cells (Fig. 1*B* and *SI Appendix*, Fig. 1 *A* and *B*). Viral SARS-CoV-2 E RNA was also detected in cultures by qRT-PCR (Fig. 1*C*). To evaluate the capacity for propagation of SARS-CoV-2 in infected primary astrocytes, we utilized plaque assays. We collected the supernatant from astrocyte cultures 3 d after exposure to SARS-CoV-2 and cultured Vero cells in this conditioned medium. We identified infection in Vero cells 3 d after onset of culturing, confirming SARS-CoV-2 not only infects, but also propagates in cortical astrocytes (Fig. 1*C*). Immature astrocytes expressing HOPX and PAX6, as well as maturing astrocytes expressing S100B and GLAST, demonstrated high infectivity (Fig. 1*D* and *SI Appendix*, Fig. 2*B*). Despite efficient infection in astrocytes labeled by a range of markers, we observed minimal infection of other cell types present in the developing cortex—in particular, <8% of infected cells were NEUN+ neurons, and <11% were KI67+ dividing cells (Fig. 1*E* and *SI Appendix*, Fig. 1*C*). Progenitor cells of the cortical excitatory neural lineage, including neurogenic radial glia and intermediate progenitor cells, were rarely infected, confirming an infection bias heavily weighted toward astroglial cells over neurons (*SI Appendix*, Fig. 2*A*). Other glial cell types, including OLIG2+ oligodendrocyte precursor cells and IBA1+ microglia, also demonstrated minimal capacity for infection (*SI Appendix*, Fig. 2*C*).

To evaluate a potential route of viral entry to the brain, we explored infection in vascular cell types in primary cortical samples. Developing blood vessels are composed of an inner layer of endothelial cells (expressing CD31 and CD34) and an exterior layer of mural cells (expressing PDGFR-β) (*SI Appendix*, Fig. 2*D*). Approximately 13% of infected cells (dsRNA+ or N+) were CD31+ endothelial cells, and 18% were PDGFR-β+ mural cells, indicating a tropism of SARS-CoV-2 infection for vascular cell types. The proportion of infected vascular cells, consisting of small blood vessels located in the SVZ, was considerably lower than the proportion of infected cortical astrocytes. Additionally, vascular units, many of which were surrounded by GFAP+ astrocytes forming the blood brain barrier, express a SARS-CoV-2 entry factor, ACE2 (*SI Appendix*, Fig. 2*D*). Astrocytes adjacent to infected vasculature were also frequently infected (*SI Appendix*, Fig. 2*D*), suggesting a potential hematologic means of viral spread in the cortex.

### SARS-CoV-2 Infects Astrocytes in Gliogenic Cortical Organoids.

**Fig. 3.**
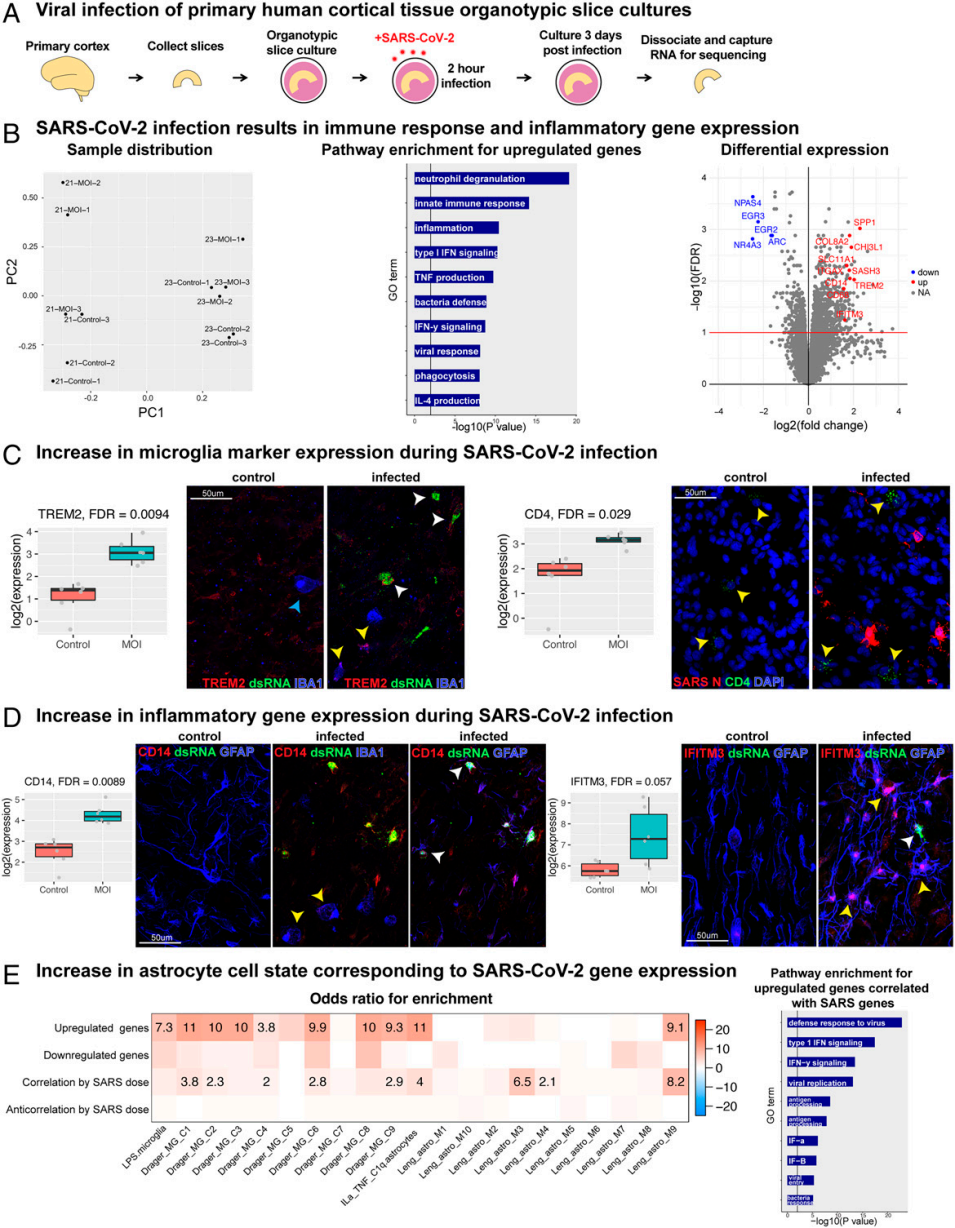
RNA-seq reveals an inflammatory response in human cortex after SARS-CoV-2 infection. (*A*) Primary organotypic cortical cultures from GW21 and GW23 were exposed to SARS-CoV-2 for 2 h and processed for RNA-seq 72 h later (three replicates for infection and control for each individual). (*B*) (*Left*) PC analysis revealed the first axis of variation as primarily due to age, while the second was related to SARS-CoV-2 infection. PC1 explained 15% of the variance and PC2 10% of the total variance. (*Middle*) Pathway enrichment analysis using Gene Ontology. A set of 22 genes are upregulated based on FDR < 0.1 and at least a 2-fold increase (100% increase or doubling) of normal expression. Upregulated genes in infected samples correspond to immune response, inflammation, neutrophil degranulation, and cytokine signaling. (*Right*) Volcano plot of significance versus effect size, with line drawn at FDR < 10%. The top downregulated genes are associated with transcription and cell growth (blue), and selected upregulated genes associated with inflammation, immune response, and microglia activation (red) are labeled. (*C*) We observe an increase in microglia markers TREM2 and CD4 expression after infection. Box plots contain expression values for the gene of interest (TREM2: FDR < 0.0094, CD4: FDR < 0.029, blue arrow: microglia without TREM2, yellow arrows: microglia with TREM2 or CD4, white arrow: dsRNA+-infected cell with TREM2). (*D*) There is an increase in inflammatory gene expression, including CD14 (FDR < 0.0089) and IFITM3 (FDR < 0.057), after infection. Protein staining indicates CD14 is increased in IBA1+ microglia (yellow arrow) and infected dsRNA+GFAP+ astrocytes (white arrow). IFITM3 is increased in infected (yellow arrow) and uninfected (white arrow) astrocytes. (*E*) There is an increase in astrocyte reactivity state corresponding to infection-related genes. Cell state enrichment analysis of differentially expressed genes from *B* and SARS-CoV-2 gene expression dose-responsive genes were compared with publicly available gene sets of activated microglia (24) and reactive astrocyte (25) cell states. Gene Ontology plots show an upregulated set of 918 genes correlated with SARS-CoV-2 dose based on FDR < 0.1 and at least a 2-fold increase of normal expression (GeneOntology up). When evaluating cell state by SARS-CoV-2 dose, our dataset most highly corresponds to an activated astrocyte population with high NF-κB signaling. Pathway enrichment of our viral dose-related dataset indicates genes that regulate viral defense, cytokine signaling, and immune response.

To evaluate the capacity for infection over a range of developmental timepoints and in a variety of cortical cell types, we utilized cortical organoids exposed to SARS-CoV-2. Organoids were infected at stages of early neurogenesis (week 5), peak neurogenesis (week 10), early gliogenesis (week 16), and peak gliogenesis (week 22) (Fig. 2*A*) (23). We observed that organoids from neurogenic and early gliogenic stages were rarely infected, and the infrequently infected cells did not express markers of SOX2+ cortical progenitors, NEUN+ neurons, or glial cells, suggesting off-target or noncortical lineages (Fig. 2*B* and *SI Appendix*, Fig. 3*A*). At week 16, we observed infection of a few GFAP+ cells in one stem cell line, but infection was absent in a second stem cell line at the same timepoint. Overall, we found rare viral N+ and no dsRNA+ cells at neurogenic stages in organoids, indicating low levels of entry without evidence of replication or productive infection (Fig. 2*C* and *SI Appendix*, Fig. 3*A*). However, by week 22 of differentiation—a stage when significant gliogenesis has occurred in our organoids—GFAP+AQP4+ astrocytes were robustly infected, consistent with our results in primary tissue samples. NEUN+ cortical neurons were rarely infected (Fig. 2 *D* and *E* and *SI Appendix*, Fig. 3 *B* and *C*). Together, experiments utilizing primary and organoid cortical cultures suggest SARS-CoV-2 can readily infect, replicate, and propagate in astrocytes in the developing human cortex.

### SARS-CoV-2 Infection Increases Immune Response and Inflammatory Gene Signatures.

**Fig. 4.**
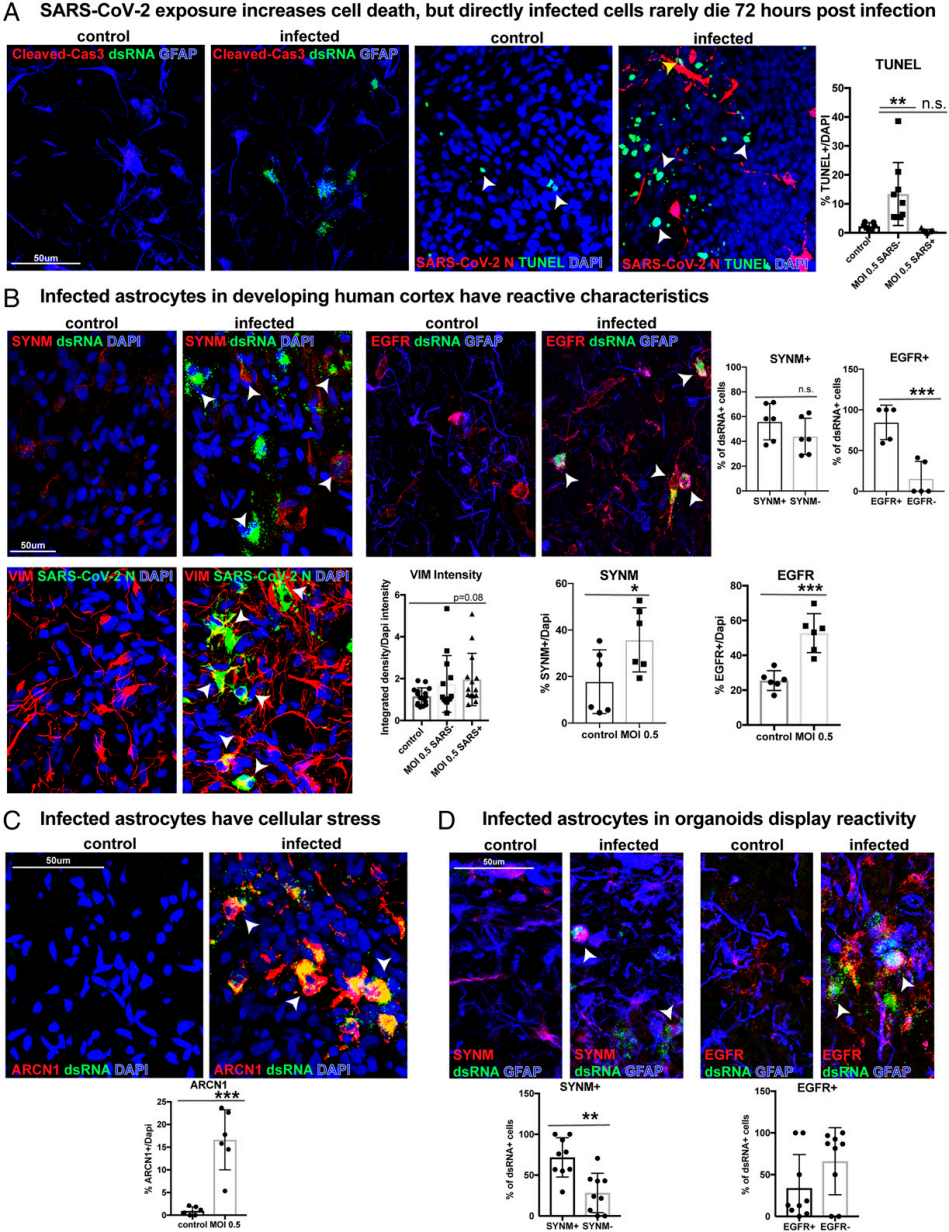
SARS-CoV-2 infection increases reactivity in cortical astrocytes. (*A*) In slice cultures exposed to SARS-CoV-2, we do not observe an increase in cleaved caspase-3 after infection. We observe an overall increase in the number of TUNEL+ cells within the infected culture (white arrow). However, the number of TUNEL+ SARS-CoV-2 N+-infected cells (yellow arrow) is not significantly different from control (ordinary one-way ANOVA with multiple comparisons: **P < 0.008, error bars represent SD, *n* = 3 biological samples from stages GW 19, 22, 23 from six technical replicates). (*B*) Infected astrocytes in the developing human cortex have reactive characteristics. Reactive markers SYNM and EGFR increase after infection compared to control, indicating an increase in reactivity postinfection (unpaired Student’s *t* test: SYNM: *P < 0.05, EGFR: ***P < 0.0003; error bars represent SD). More than 55% of infected primary astrocytes express SYNM, and 85% express EGFR (unpaired Student’s *t* test: SYNM *P* = 0.19, EGFR ***P < 0.0009, error bars represent SD, *n* = 3 biological samples from stages GW 19, 22, 23 from five technical replicates). The reactive astrocyte marker, VIM, also trends toward increased intensity in infected cells (ordinary one-way ANOVA with multiple comparisons: *P* = 0.08, error bars represent SD, *n* = 3 biological samples from 12 technical replicates). (*C*) Infected samples have a 15% increase in the ER stress marker, ARCN1. All ARCN1+ cells coexpress SARS-CoV-2 dsRNA (unpaired Student’s *t* test: control vs. MOI 0.5, ***P < 0.0002, error bars represent SD, *n* = 3 biological samples from stages GW 19, 22, 23 from six technical replicates). (*D*) Infected astrocytes in organoids display reactivity. In total, 72% of infected organoid cells express SYNM, and about one-third express EGFR (unpaired Student’s *t* test: SYNM **P < 0.0014, EGFR *P* = 0.109, error bars represent SD, *n* = 2 cell lines from four technical replicates).

To assess the impact of SARS-CoV-2 infection on cortical tissue in an unbiased manner, we performed bulk RNA sequencing (RNA-seq) of organotypic primary cultures after exposure to SARS-CoV-2 (Fig. 3*A*). Principal-component (PC) analysis and hierarchical clustering revealed a clear transcriptomic signature of SARS-CoV-2 infection (Fig. 3 *B*, *Left* and *SI Appendix*, Fig. 4*A*). Differential gene expression analysis revealed an increase in expression for genes involved in inflammation, the innate immune response, and the defense response to viral infection (Fig. 3 *B*, *Middle, **SI Appendix*, Table 1). Relatively fewer genes were downregulated, but those that decreased were involved in fundamental cellular processes including cholesterol biosynthesis, calcium transport, cell size, and morphology (*SI Appendix*, Fig. 4*A*).

**Fig. 5.**
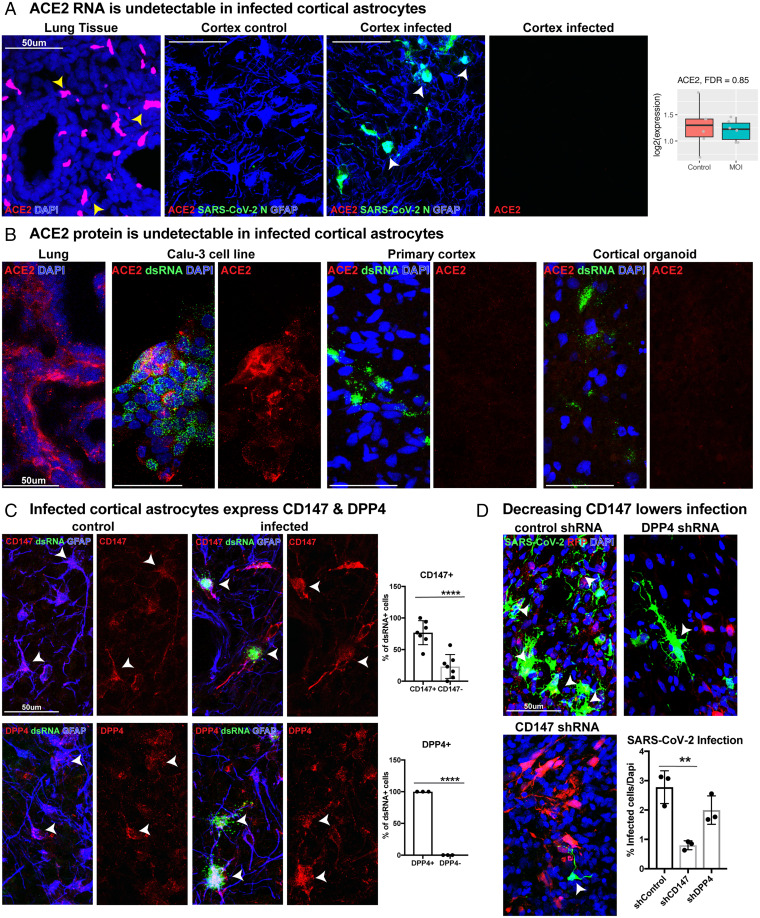
Coronavirus receptors CD147 and DPP4, but not ACE2, are expressed in human cortex. (*A*) *In situ* hybridization for ACE2 RNA was performed in developing lung tissue (positive control) and developing cortical tissue. While ACE2 is highly expressed in the lung (yellow arrowheads), ACE2 was not detected in human cortical tissue or in infected cortical astrocytes (white arrowheads). ACE2 RNA also does not increase after infection (*P* = 0.85). (*B*) ACE2 protein is undetectable in cortical astrocytes. Immunohistochemistry for ACE2 protein was performed on lung tissue and a lung cell line, Calu-3, confirming efficient protein detection. We find no observable ACE2 protein in primary cortical tissue, cortical organoids, or infected cells in either sample type. (*C*) Cortical astrocytes express coronavirus receptors CD147 and DPP4. In total, 76% of infected dsRNA+ GFAP+ astrocytes express the coronavirus receptor CD147, and 100% express DPP4 (unpaired Student’s *t* test: CD147 ***P < 0.0002, DPP4 ****P < 0.0001, error bars represent SD, *n* = 3 biological samples from stages GW 19, 22, 23 from four technical replicates). (*D*) Organotypic cultures were treated with lentivirus containing shRNAs against CD147 or DPP4 to knock down CD147 or DPP4, respectively. After decreasing the abundance of CD147, there was a significant reduction in infection rate (ordinary one-way ANOVA: control vs. CD147 **P < 0.034, control vs. DPP4 *P* = 0.15, error bars represent SD, *n* = 3 technical replicates from two biological samples).

Among the top upregulated genes were those implicated in inflammation, injury response, and microglial activation (DGE_VolcanoPlot) (Fig. 3*B*, *Right*). Microglia markers and activation genes—including TREM2, CD4, SPP1, ITGAX—were also increased in infected cortical tissue (Fig. 3*C* and *SI Appendix*, Figs. 4 *B* and *C* and 5*A*). Additionally, many indicators of inflammation, cytokine secretion, and immune activation had increased expression (Fig. 3*D* and *SI Appendix*, Fig. 4 *B* and *C* and 5*B*). In addition to an increase in inflammatory and reactivity gene expression in microglia, there was an increased abundance of CD14, IFITM3, and TREM2 protein in infected astrocytes (Fig. 3 *C* and *D*, white arrowheads).

Genes upregulated and downregulated in the differential expression analysis of infection compared to controls (Fig. 3*B*) represent both direct and indirect effects of SARS-CoV-2 infection. To refine our evaluation of how SARS-CoV-2 infection impacts microglia and astrocytes, we sought to compare the gene signatures most associated with SARS-CoV-2 infection. Focusing on infected samples only, we identified the top genes with high correlation (*R* > 0.8) and anti-correlation (R < −0.8) to SARS-CoV-2 E and N gene levels (*Materials and Methods*). The top genes were involved in viral response and entry, type I interferon (IFN) signaling, IFN-gamma signaling, Major Histocompatibility Complex (MHC) activation, and interleukin 4 signaling (Fig. 3 *E*, *Right*).

### SARS-CoV-2 Infection Increases Reactivity in Cortical Astrocytes.

To further understand the role of activated microglia and reactive astrocytes, we compared genes differentially expressed postinfection, which were upregulated in parallel with the quantity of SARS-CoV-2 E and N expression, to gene sets representing reactive cell states (Fig. 3*E*) (25, 24). Although there was enrichment for microglial and astrocyte activation in the gene set upregulated in infection, genes associated with high SARS-CoV-2 E and N were overwhelmingly enriched in two reactive astrocyte states that appear to be downstream of Nuclear factor kappa B (NF-κB) signaling. There was relatively lower correlation to microglia. These results suggest that while an indirect effect of SARS-CoV-2 infection impacts both microglia and astrocytes, a direct, dosage-sensitive effect of SARS-CoV-2 is observed specifically in reactive astrocytes. We, therefore, focused our investigation on the impact of infection on cortical astrocytes.

**Fig. 6.**
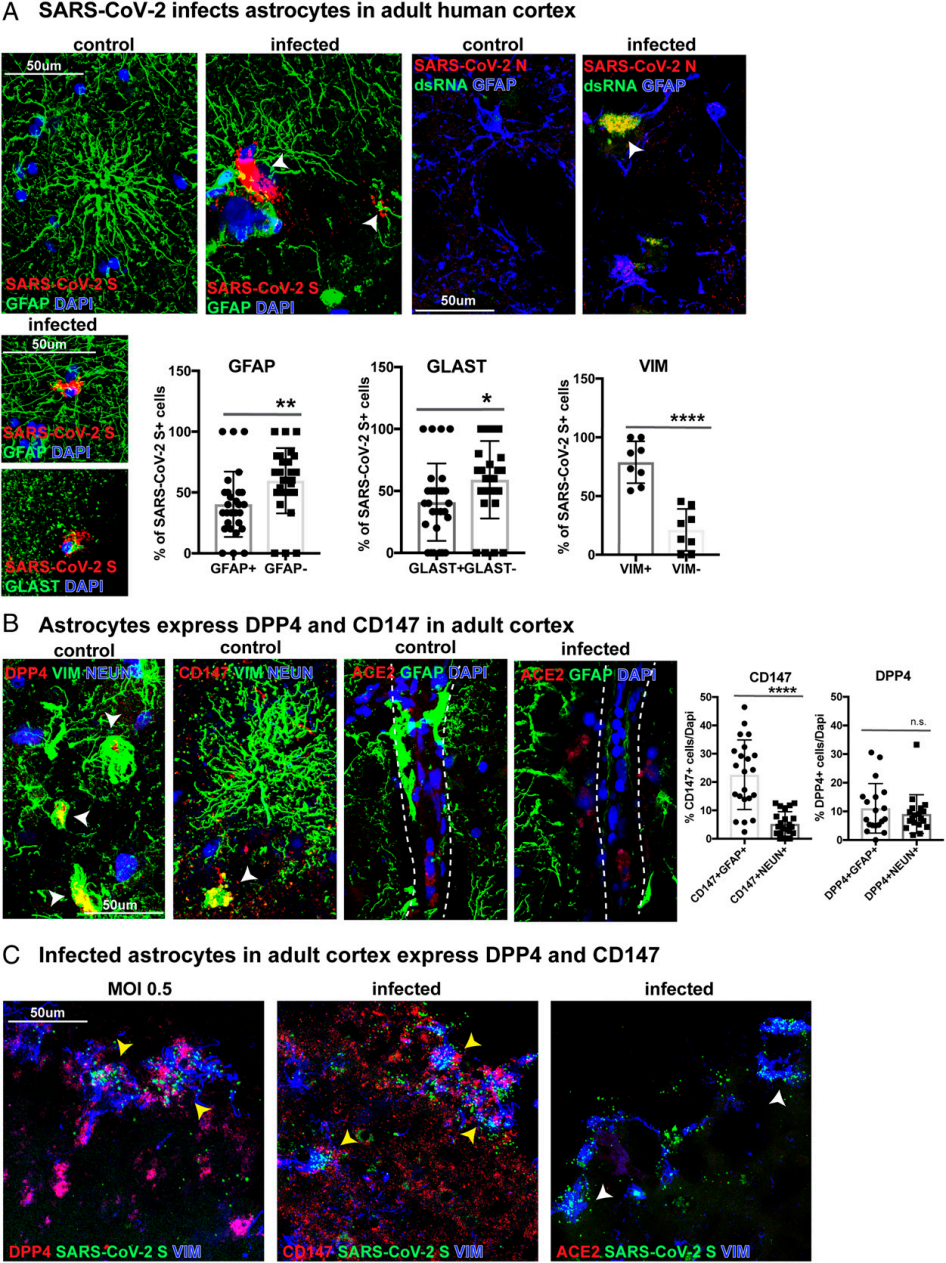
Astrocytes in the adult human cortex are vulnerable to SARS-CoV-2 infection. (*A*) Surgically resected adult human cortical tissue from 19 and 34 y of age was acutely sectioned and exposed to SARS-CoV-2 for 2 h and cultured for 48 to 72 h before collection. GFAP+, GLAST+, or VIM+ astrocytes in the adult cortex were infected and labeled by SARS-CoV-2 spike (S) riboprobe or SARS-CoV-2 nucleocapsid (N) antibody. Of the infected cells, 40% were GFAP+, 41% were GLAST+, and 79% were VIM+, indicating different populations of astrocytes are vulnerable to infection (unpaired Student’s *t* test, GFAP **P < 0.01, GLAST *P < 0.036, VIM ****P < 0.0001, *n* = 2 biological samples from four technical replicates). (*B*) In the adult cortex, coronavirus receptors DPP4 and CD147 are expressed on VIM+ astrocytes (white arrows), while ACE2 is localized to blood vessels. There is a greater abundance of CD147 in GFAP+ astrocytes than NEUN+ neurons, while DPP4 is expressed in both cell types (unpaired Student’s *t* test, CD147+GFAP+ vs. CD147+NEUN+ ****P < 0.0001, DPP4+GFAP+ vs. DPP4+NEUN+ *P* = 0.424). (*C*) Infected SARS-CoV-2 S+ VIM+ astrocytes in the adult cortex express DPP4 and CD147 (yellow arrows), but ACE2 is not observed in infected cells (white arrows).

To assess the functional consequences of SARS-CoV-2 infection and inflammation on cortical astrocytes, we assayed the abundance of the apoptotic markers cleaved-caspase-3 and terminal deoxynucleotidyl transferase dUTP nick end labeling (TUNEL). We observed no increase in apoptosis in infected dsRNA+ or SARS-CoV-2 N+ GFAP+ primary astrocytes 72 h postinfection (Fig. 4*A* and *SI Appendix*, Fig. 6*A*). However, there was an increase in the overall number of TUNEL+ apoptotic cells in cultures exposed to SARS-CoV-2 (Fig. 3*D*). Although we observed no change in astrocyte cell death, there was an overall decrease in the number of neurons (*SI Appendix*, Fig. 7*A*), likely as a consequence of cell nonautonomous inflammatory effects throughout the culture. However, there were no global changes in expression of neuronal or astrocyte marker genes in bulk RNA-seq datasets (*SI Appendix*, Fig. 7 *B* and *C*).

Due to the increase in cell death in infected cultures, we sought to evaluate whether infected astrocytes or adjacently located uninfected astrocytes have atypical characteristics, including increased reactivity or activated growth factor signaling, common after SARS-CoV-2 infection (26). More than 50% of infected cells expressed the reactive marker Synemin (SYNM) (27, 28), and the number of SYNM+ cells increased by 20% postinfection compared to controls (Fig. 4*B*). Another marker of reactivity, Vimentin (VIM) (29), trended toward increased intensity after SARS-CoV-2 exposure in infected astrocytes (Fig. 4*B* and *SI Appendix*, Fig. 6*B*). Aberrant activation of growth receptor signaling can also result after coronavirus infection, and inhibition of growth factor receptors can prevent SARS-CoV-2 replication (26). We evaluated the presence of epidermal growth factor receptor (EGFR), which is involved in the regulation of inflammatory states and is expressed by some glial precursor cell types (30, 31). We observed that more than 75% of infected cells expressed EGFR, and total abundance of EGFR doubled after SARS-CoV-2 infection compared to control conditions (Fig. 4*B*). Additionally, there are a greater number of SYNM+ reactive astrocytes that are uninfected within cultures exposed to SARS-CoV-2 (*SI Appendix*, Fig. 7*D*). Organoid-derived astrocytes also exhibit some reactive characteristics in response to infection, with ∼70% of organoid-derived infected cells expressing SYNM and 33% expressing EGFR (Fig. 4*D*). These findings indicate both an increase in a reactive-like state postinfection as well as a non-cell-autonomous impact on neighboring astrocytes and neurons after SARS-CoV-2 infection (Fig. 4*B*).

In recent studies of adult postmortem samples, infected astrocytes were observed to have impaired protein folding, translation initiation, and metabolic function and an increase in inflammation and gliosis gene expression (8, 32). We investigated the impact of SARS-CoV-2 on cellular stress by evaluating the abundance of endoplasmic reticulum stress and the unfolded protein response gene ARCN1. ARCN1 is lowly expressed in developing human cortex tissue, but after infection by SARS-CoV-2, we found a 15% increase in expression, exclusively in infected cells coexpressing dsRNA (Fig. 4*C* and *SI Appendix*, Fig. 6*C*).

### The Canonical SARS-CoV-2 Receptor, ACE2, Is Not Detected in Cortical Astrocytes.

The robust capacity for infection in cortical astrocytes across model systems highlights questions concerning the mechanism mediating viral tropism. We began by exploring the abundance of the ACE2 receptor in cortical tissues and particularly in astrocytes, as ACE2 is the canonical entry factor for SARS-CoV-2. Reanalysis of our published single-cell RNA-seq (scRNA-seq) dataset of primary human cortex during development demonstrated essentially null expression in cortical cells, including progenitors, neurons, and astrocytes (*SI Appendix*, Fig. 8*A*) (hgwdev-max.soe.ucsc.edu/tsneViewer/dev3/) (33). We further compared bulk RNA expression of ACE2 in developing human cortex samples, cortical organoids, and adult human cortex tissue, as well as in developing lung tissue. We observed very little expression in cortical tissue compared to the lung (*SI Appendix*, Fig. 8*A*). To evaluate ACE2 RNA abundance in cortical tissue, we utilized fluorescent *in situ* hybridization of an ACE2 RNA probe, using lung tissue as a positive control (Fig. 5*A*). Although we observed robust expression of ACE2 throughout the lung, as well as modest expression in vascular cells (*SI Appendix*, Fig. 2*D*), it was absent in cortical astrocytes. To determine if a subset of astrocytes might express or upregulate ACE2 postinfection, we assayed ACE2 abundance in infected cortical samples and observed no upregulation of ACE2 in our RNA-seq dataset or in SARS-CoV-2 N+ GFAP+-infected astrocytes (Fig. 5*A*).

As there can be a divergence between RNA expression and protein abundance, we examined immunolabeling for ACE2 in infected cortical tissue and organoids. Although ACE2 was readily detected in the lung and the Calu-3 lung cell line, we detected no ACE2 expression across cortical samples or in dsRNA+-infected cortical cells in primary cortical tissue or organoids (Fig. 5*B*). We were also unable to detect ACE2 protein by western blot in cortical tissue or in normal human astrocyte cultures (*SI Appendix*, Fig. 8*B*). However, in late-stage organoids, bulk RNA and protein assays indicated low expression of ACE2. This may reflect differences in gene expression in organoid cells compared to primary tissue or ACE2 expression in rare and perhaps off-target populations that can be present in cortical organoids (23) but are not present in primary cortical tissue of comparable developmental stages (*SI Appendix*, Fig. 8 *A* and *B*).

NRP1 is a potential candidate entry factor for infection, as it has been demonstrated to be a SARS-CoV-2 host factor (34). We observed NRP1 RNA expression in primary tissue, specifically in cortical neurons—a population, however, where we observed minimal infection (*SI Appendix*, Fig. 8*A*). When we evaluated NRP1 protein abundance, we similarly observed membrane-bound staining in neurons in the cortical plate in primary tissue, but none in dsRNA+-infected cells (*SI Appendix*, Fig. 8*C*).

Recent studies have demonstrated that comediating factors are involved in SARS-CoV-2 infection. Expression of specific receptors, restriction enzymes, and proteases may be vital for mediation of SARS-CoV-2 infection in collaboration with, or perhaps independent of, ACE2 (34–37). We evaluated whether the extracellular glycoproteins and coronavirus receptors DPP4 and BSG/CD147 are expressed in our primary developing human scRNA-seq dataset. We observed broad expression in a variety of cell types and high expression in bulk RNA datasets across cortical organoids, developing human cortex, and adult cortex samples (*SI Appendix*, Figs. 8*A* and 9*B*). Additionally, SARS-CoV proteases TMPRSS2, FURIN, and CTSB express modest RNA levels across cortical tissue samples, but infected astrocytes have abundant protein of all three proteases (*SI Appendix*, Fig. 10). SARS-CoV-2 restriction factors are also present. LY6E is expressed in a variety of cortical cell types across development, while IFITM1 RNA expression is most robust in adult cortex (*SI Appendix*, Fig. 8*D*). Despite the absence of ACE2, other receptors, proteases, and restriction factors required to mediate viral tropism are present in cortical tissue.

### Coronavirus Receptors CD147 and DPP4 Can Mediate SARS-CoV-2 Infection in Astrocytes.

Given the absence of ACE2 and NRP1 in cortical astrocytes as well as the presence of the coronavirus entry factors DPP4 and BSG/CD147, we investigated the abundance of CD147 and DPP4 in our infected primary tissue samples. In dsRNA+-infected cells, we observed 100% colabeling with DPP4 and 77% colabeling with CD147+ (Fig. 5*C* and *SI Appendix*, Fig. 11*A*). Although cortical organoids have lower receptor abundance than primary tissue, infected organoid cells also demonstrated coexpression of DPP4 and CD147 (*SI Appendix*, Fig. 12*A*).

Due to the abundance of CD147 and DPP4 and their known role as mediators of neuroinflammatory and reactive states in astrocytes (38), we evaluated whether CD147 and DPP4 could mediate cortical astrocyte tropism. We designed lentiviruses containing short hairpin RNAs (shRNAs) directed against CD147, DPP4, or ACE2 and validated knockdown efficiency in cortical astrocytes differentiated from primary glial progenitors (39) (*SI Appendix*, Fig. 12*B*). Primary organotypic cortical slice cultures were treated with lentiviral shRNAs for 5 d prior to exposure to SARS-CoV-2 for a duration of 2 h. Three days after SARS-CoV-2 exposure, astrocytes treated with the control scramble-red fluorescent protein (RFP) lentivirus displayed robust infection by SARS-CoV-2 (Fig. 5*D*). When slices were pretreated with a potent shRNA against CD147, there was a significant reduction in SARS-CoV-2 infection (Fig. 5*D*). However, due to the high levels of DPP4 expression (*SI Appendix*, Fig. 8*B*) and established translational control of protein abundance as a response to inflammation (40), the efficiency of DPP4 knockdown was significantly less than other receptors (*SI Appendix*, Fig. 12*B*), and the reduction in knockdown did not reach significance (Fig. 5*D*) (40). Surprisingly, despite the lack of detectable ACE2 RNA or protein in cortical astrocytes across a variety of assays, when ACE2 was efficiently knocked down, there was also an overall reduction in infection (*SI Appendix*, Fig. 12*G*). We, therefore, cannot rule out a role for ACE2 in astrocyte infection, but one possible interpretation is that the presence of ACE2 in vascular cells in the organotypic slice cultures (*SI Appendix*, Fig. 2*D*) (20) may be involved in initial infection with subsequent consequences on ACE2-lacking cell types.

To probe the functional role of DPP4 in infectivity, organotypic slice cultures were treated with the DPP4 inhibitor Vildagliptin for 24 h prior to and throughout the duration of infection. As before, there was robust infection in GFAP+ astrocytes in virally exposed samples after 72 h. However, in tissue samples treated with the DPP4 inhibitor, there was a 35% reduction in the number of SARS-CoV-2 N+ cells and a 60% reduction in dsRNA+ cells (*SI Appendix*, Fig. 12*E*). Additionally, there was a 70% reduction in the number of cells expressing ARCN1 after DPP4 inhibition, indicating decreased activation of cellular stress.

To determine whether CD147 and DPP4 are sufficient for viral entry in the absence of ACE2, as has been identified for CD147 in other systems (41), we performed a gain-of-function study by overexpressing CD147 or DPP4 using lentiviruses in week 5 cortical organoids. At this stage, organoids lack ACE2 (*SI Appendix*, Fig. 13*A*), do not demonstrate viral tropism (Fig. 2*B*), and do not contain cortical astrocytes (23), thus providing a model to test the sufficiency of these receptors to mediate infection (*SI Appendix*, Fig. 13*B*). We exposed the CD147- or DPP4-expressing organoids to SARS-CoV-2 for 2 h. We observed a significant increase in SARS-CoV-2 N+ cells with DPP4 overexpression and an increase in dsRNA+ cells in both DPP4- and CD147-overexpressing conditions (*SI Appendix*, Fig. 13*C*). Taken together with the loss of function and inhibition studies, these findings indicate that DPP4 and CD147 are sufficient to mediate SARS-CoV-2 infection and suggest that DPP4 may promote viral entry, while CD147 may promote viral replication.

### SARS-CoV-2 Infects Astrocytes in Primary Adult Human Cortex.

Although there are reports of gestational and neonatal transmission with neurological consequences in cases of severe infection in preterm and newborn infants (42–44), most COVID-19-related neurological symptoms occur in adults. As both organoids and preterm tissue samples are reflective of developmental stages, we sought to determine whether astrocyte vulnerability may extend to postnatal and adult life. We obtained donated cortical samples from patients 19 and 34 y of age undergoing surgical resection of epileptic tissue. The samples represented healthy cortical tissue that needed to be removed to provide surgical access. Cortical slice cultures were exposed to SARS-CoV-2 for 2 h and collected for evaluation after 48 to 72 h. We observed that different populations of mature astrocytes expressing GFAP+, GLAST+, or VIM+ (*SI Appendix*, Fig. 9 *A* and *C*) were infected by SARS-CoV-2, coexpressing SARS-CoV-2 S and N (Fig. 6*A*). We found that GFAP+ adult cortical astrocytes, similar to developing astrocytes, express both CD147 and DPP4. While we observed higher expression of CD147 in GFAP+ astrocytes than NEUN+ neurons, DPP4 had similar levels of expression in both cell types (Fig. 6*B* and *SI Appendix*, Figs. 9*B* and 14). We observed ACE2 expression along blood vessels but found no expression in adult cortical cell types (Fig. 6*B* and *SI Appendix*, Fig. 9 *B* and *D*). Moreover, infected SARS-CoV-2 S+ VIM+ astrocytes expressed DPP4 and CD147 but not ACE2 (Fig. 6*C*). The high abundance of VIM in infected cells suggests both specific astrocyte tropism as well as reactivity in adult tissue. Together with our observations in infected organoids and developing tissue, these data suggest tropism of SARS-CoV-2 for astrocytes in both developing and adult human cortex. Furthermore, the capacity for viral propagation can occur independent of ACE2 and is mediated, in part, by CD147 and DPP4.

## Discussion

Our study exploring neural tropism of SARS-CoV-2 demonstrates that human cortical astrocytes can be directly infected and display downstream cellular stress and glial reactivity. We additionally demonstrate that vascular cell types, including endothelial and mural cells, can be infected. Vascular cells are positioned adjacent to astrocytes in blood–brain barrier formation, and their close proximity provides a potential mode of entry for infection, particularly given the high levels of ACE2 expression in vascular cell types and microvascular injury that can occur as a consequence of COVID-19 (16, 20). Although in the current study cortical cultures were exposed to SARS-CoV-2 virus by bath application, rather than through systemic exposure, the results highlight specific astrocyte vulnerability and tropism. Since astrocytes are a key support cell that regulate myriad vital functions in the developing and adult brain, the strong tropism of the SARS-CoV-2 virus has implications for neurological function. Astrocytes regulate neurotransmitter concentration and reuptake for appropriate synaptic communication, mediate blood–brain barrier function, and regulate neural metabolism and inflammation (45). Disorders of astrocyte development results in increased cellular stress and autophagy; changes to morphology; inability to regulate concentration of neurotransmitters and ions for appropriate synaptic activity; and decoupling of astrocytes to one another, blood vessels, and the neuronal microenvironment (45). In the adult and aged brain, astrocytes become reactive in inflammatory, ischemic, and neurodegenerative disease states, which furthers neurological damage (46). We observe that a short exposure to infection partly recapitulates transcriptional changes observed in reactive astrocytes and microglia (8).

In this study, we treated primary developing and adult cortical tissue and cortical organoids with a low liter of SARS-CoV-2 and observed minimal direct infection in neurons after 72 h. Despite widespread inflammation and scattered dead cells, we observed SARS-CoV-2 tropism primarily in astrocytes. Our findings demonstrate increased reactivity not only in infected astrocytes, but in neighboring astrocytes as well, suggesting that SARS-CoV-2 infection may lead to both direct and indirect impairment of cellular function. We additionally observed cell death in uninfected cells with a corresponding decrease in neuronal numbers, suggesting indirect effects on neuron viability. We observed only rare neuronal infection, consistent with studies of postmortem patient neural tissue samples that show inconsistent evidence that neurons may be susceptible to SARS-CoV-2 infection (47–49). Higher viral titers or prolonged exposure to the virus may make neurons more permissible to tropism and may explain differing findings regarding cortical neuron tropism. Future studies exploring longer viral exposure and the impact of inflammation and cellular reactivity may provide insight into long-term consequences to neuronal viability and function. Outside of the possibility of viral tropism and CNS entry, systemic inflammation and immune activation play a clear role in CNS pathology. Studies of pathological samples have demonstrated that even in the absence of viral RNA in neural tissue, changes in astrocyte and microglia states can be observed (8, 9). Moreover, individuals with neurological phenotypes demonstrate CNS-specific T cell and B cell activation (9). Patient data suggest that CNS inflammation alone may produce neurological dysfunction and symptomatology.

Although SARS-CoV-2 infection of airway epithelia and lung parenchyma is predominantly mediated by the ACE2 receptor, this study and others provide evidence that alternative coronavirus receptors may participate in entry and viral replication (34, 41). The capacity of SARS-CoV-2 to infect cell types that do not demonstrate apparent expression of ACE2 and instead express other extracellular glycoproteins suggests a capacity to infect a variety of cell types using a range of receptors. Our findings suggest that CD147 is necessary and DPP4 is sufficient to mediate SARS-CoV-2 infection in cortical astrocytes. Both DPP4 and CD147 are known to increase in response to inflammation in reactive astrocytes (38, 40, 50), and both proteins appear to be upregulated in infected astrocytes. However, in the absence of infection, neither CD147 nor DPP4 abundance changes in cortical slices over time, markers of reactivity do not change from baseline, and inhibition of these receptors does not affect reactivity states (*SI Appendix*, Fig. 12 *C*, *D*, and *F*), indicating that reactive astrocytes with aberrant receptor abundance are a postinfection response. However, DPP4 and CD147 may not be the sole receptors that mediate infection since a low level of infection can persist after both are inhibited. Moreover, although ACE2 RNA and protein are undetectable in infected cells, it is possible that a less-than-detectable quantity of ACE2 may participate in infection, as ACE2 knockdown was able to decrease infection. Exploration for additional novel SARS-CoV-2 receptors—such as VIM (51), which is expressed in cortical astrocytes, is involved in reactivity, and increases postinfection, may identify additional entry factors utilized by the virus.

In this study, we analyzed primary cortical tissue, in parallel with organoid models, at a range of developmental and adult timepoints. While cerebral organoid models may have limitations in terms of the diversity and fidelity of neural cell types they contain (23), our studies indicate that organoid-derived astrocytes are preferentially infected by SARS-CoV-2, similar to our findings in primary human cortical tissue. Several studies have reported SARS-CoV-2 tropism, mainly mediated through ACE2, in a variety of neural and glial cell types in organoids (13, 14, 19–21, 52–54). While we did not detect ACE2 in primary tissue, we observed variable ACE2, DPP4, and CD147 expression in organoids at corresponding stages. Conflicting reports on the prevalence and identity of infected neural cell types based on studies utilizing organoid models may relate to ectopic ACE2 expression, variability of gene expression across different stem cell lines, or expression of ACE2 in cell types of different regional identities. Additionally, organoid studies have typically reported results of infection at one discrete timepoint, often during early neurogenic periods, when vulnerable cell types may not be present. Here, we utilized organoids from several stages of development treated with a low titer of SARS-CoV-2 and observed robust infection and viral replication only when cortical astrocytes were present, reflective of our findings in human primary cortical tissue at both developing and adult ages.

The results of our adult *in vitro* infection and RNA expression analyses demonstrated moderate expression of coronavirus receptors CD147 and DPP4 in the adult cortex. Future studies exploring postmortem patient neural tissues will determine how frequently astrocytes may be targeted by SARS-CoV-2 in infected infants, children, adults, and aged adults. These studies will assist in identifying endogenous astrocyte vulnerability and, when paired with neurological data, may help correlate cell type vulnerability with neurological and neuropsychiatric outcomes. Of particular concern are recent reports of new or recurring neuropsychiatric consequences in children and adolescents whose CSF contains SARS-CoV-2 antibodies, even in the absence of symptomatic COVID-19 (3). The range of COVID-19-associated neurological symptoms including dizziness, seizures, and cognitive difficulties, may reflect the involvement of astrocytes, which are vital to global brain homeostasis and function. Further studies elucidating potential routes of CNS infection as well as strategies to block viral entry may aid in controlling transmission and help alleviate disease symptoms.

## Materials and Methods

### Summary.

Detailed methods are in *SI Appendix*. Briefly, primary organotypic slice cultures from developing (GW19-23) and adult (19, 34 y of age) human cortex and cortical organoids (weeks 5 to 24) were exposed to SARS-CoV-2 at a Multiplicity of Infection (MOI) 0.5 for 2 h. Three days later, samples were processed for immunohistochemistry and RNA-seq.

## Supplementary Material

Supplementary File

## Data Availability

Original data created for the study are available in *SI Appendix,* Table 1. RNA-seq data have been deposited in the National Center for Biotechnology Information (NCBI) dbGaP, study accession phs000989 (https://www.ncbi.nlm.nih.gov/gap/?term=1[s_discriminator]%20AND%20Arnold%20Kriegstein&report=SStudies) (55).
