# Supplementary material for: Tropism of SARS-CoV-2 for human cortical astrocytes
Source: Proc Natl Acad Sci U S A. 2022 Jul 12;119(30):e2122236119. doi: 10.1073/pnas.2122236119 (PMC9335272; doi:10.1073/pnas.2122236119)
Supplement: Supplementary File [file pnas.2122236119.sapp.pdf]

Methods

Lead contact and materials availability

Any questions and requests for resources should be directed to Arnold Kriegstein, at Arnold.Kriegstein@ucsf.edu.

Author Contributions

M.G.A., T.M., C.R.S, G.R.K, M.O. & A.R.K. designed the study and analysis. Experiments were performed by T.M., M.G.A., C.R.S., J.R., S.W., L.Z., M.K., K.M.G., T.T., Y.P., L.W., M.A.M-R., M.M., K.C.D., D.S., J.S., and E.E.C. Data analysis was performed by U.C.E., N.P., D.V., T.M., C.V.S. and M.G.A. The study was supervised by M.G.A. T.M., A.A.P., T.J.N., E.U., E.A.W., E.E.C., M.O. and A.R.K. This manuscript was prepared by M.G.A. and T.M. with input from all authors.

Competing interest statement

ARK is a co-founder, consultant, and member of the Board of Neurona Therapeutics. The other authors declare no competing interests.

Data availability

Original data created for the study is available in SI Appendix Table 1. RNAseq data will be available in dbGaP upon publication at dbGAP study accession: phs000989.

Experimental model and subject details

Pluripotent Stem Cell Lines

H1/WA01 human embryonic stem cell line
H28126 human induced pluripotent stem cell line
13234 human induced pluripotent stem cell line

Primary Human Cortex Tissue

All primary tissue samples were obtained and processed following approval 10-05113 by UCSF Gamete, Embryo and Stem Cell Research Committee (GESCR). Tissue was collected with consent from patients for research and in strict observance of legal, institutional and ethical regulations. All samples were de-identified, and no sex information is known.

Methods details

Pluripotent stem cell expansion culture

Human induced pluripotent stem cell lines, 13234 and H28126(55, 56) and the embryonic stem cell line, WA01 (H1), were expanded on growth factor-reduced Matrigel (BD)-coated six well plates. Cells were thawed in StemFlex Pro Media (Gibco) containing 10uM Rock Inhibitor Y-27632. Media was changed alternate days and lines were passaged at 70% confluency. Cells were passaged using ReLeSR^{TM} (Stem Cell Technologies) and residual cells plated again on fresh Matrigel-coated plates. All lines in this study were between passage 25-40.

PSC line authentication

All cell lines in this study were validated for pluripotency and karyotyped. Every 10 passages cells were tested for karyotypic abnormalities and validated for pluripotency markers Sox2, Nanog and Oct4. All cell lines tested negative for mycoplasma.

Cortical organoid differentiation protocol

Cortical organoids were derived using a forebrain directed differentiation protocol (55, 57). Stem cell lines were expanded and dissociated to single cells using accutase. After dissociation, cells were reconstituted in neural induction media maintaining a density of 10,000 cells per well of 96-well V-bottom low adhesion plates. First media used is GMEM-based neural induction which includes 20% Knockout Serum Replacer (KSR), 1X non-essential amino acids, 0.11mg/mL Sodium Pyruvate, 1X Penicillin-Streptomycin, 0.1mM Beta Mercaptoethanol, 5uM SB431542 and 3uM IWR1-endo. Cells were treated with 20uM Rock inhibitor Y-27632 for the first 6 days. After 18 days, organoids were transferred to 6-well low adhesion plates and moved to an orbital shaker rotating at 90 RPM and changed to DMEM/F12-based media containing 1X Glutamax, 1X N2, 1X CD Lipid Concentrate and 1X Penicillin-Streptomycin. After 35 days, organoids were moved into DMEM/F12-based media containing 10% FBS, 5ug/mL Heparin, 1X N2, 1X CD Lipid Concentrate and 0.5% Matrigel. At 70 days media was additionally supplemented with 1X B27 and Matrigel concentration increased to 1%. Throughout culture duration organoids were fed every other day. Organoids were collected for infection and RNA extraction at weeks 5, 10, 16 and 22 of differentiation.

Organotypic slice culture of developing tissue

Primary cortical tissue from GW 19-23 was maintained in artificial cerebrospinal fluid (125 mm NaCl, 2.5 mm KCl, 1 mm $MgCl_2$, 2 mm $CaCl_2$, 1.25 mm NaH_2PO_4, 25 mm $NaHCO_3$, 25 mm d-(+)-glucose) bubbled with 95% O_2/5% CO_2 until embedded in a 3.5% low melt agarose gel. Embedded tissue was acute sectioned at 350um using a vibratome (Leica) and plated on Millicell (Millipore) inserts in a 6 well tissue culture plate. Slices were cultured at the air liquid interface in media containing 32% Hanks BSS, 60% BME, 5% FBS, 1% glucose, 1% N2 and 1% Penicillin-Streptomycin-Glutamine. Slices were maintained for 7-10 days in culture at 37°C and the media changed every third day.

Organotypic slice culture of adult tissue

Cortical tissue samples were isolated from UCSF hospital patients undergoing neurosurgical operations. Patients provided written informed consent, prior to surgery, for samples to be collected and utilized for research purposes. The protocol for tissue collection was approved by the UCSF institutional review board and ethics committee. Samples were obtained from non-pathological temporal lobe tissue, resected from epilepsy patients and immediately submerged in N-Methyl D-Glucamine artificial cerebrospinal fluid (NMDG-ACSF) pre-oxygenated with carbogen gas (95% O_2/5% CO_2). NMDG ACSF was prepared as follows (in mM): 92 NMDG, 2.5 KCl, 1.25 NaH_2PO_4, 30 $NaHCO_3$, 20 HEPES, 25 glucose, 2 thiourea, 5 Na-ascorbate, 3 Na-pyruvate; (in mL) 0.5 $CaCl_2 \cdot 2H_2O$, and 10 $MgSO_4 \cdot 7H_2O$ and titrated to pH 7.3–7.4 with 7 mL +/- 0.5 mL of 5 M hydrochloric acid per liter with an osmolality was 300–305 mOsmoles/Kg. Constant

carbonation of ACSF containing tissue was maintained during transport from hospital to lab where tissue was immediately acutely sectioned on a Leica vibratome embedded in 1.75% low melting agarose at 300um. Tissue was then placed on Millicell culture inserts and cultured at the air-liquid interface in media consisting of 95 mL MilliQ H_2O, 840 mg MEM Eagle medium, Hanks salts + 2mM L-glutamine, 18 mg Ascorbic Acid, 3 mL 1M HEPES, 1.68 mL $NaHCO_3$ (892.75 mM solution), 1.126 mL D-Glucose (1.11M solution), 0.5 mL Penicillin/Streptomycin, 0.25 mL GlutaMax, 100 uL 2M $MgSO_4.7H_2O$, 50 µL 2M stock $CaCl_2.2H_2O$, 50 µL insulin from bovine pancreas (10 mg/mL), and 20 mL heat inactivated horse serum. Slices were maintained for 5 days in culture at 37°C and the media changed every third day.

Normal Human Astrocytes (NHA) and Vero cell culture

Normal Human Astrocytes (NHA) were cultured in DMEM+10%FBS and 1%PenStrep. Alternately, they were also cultured with BMP4 and CNTF. We tested both cultures by Western blotting for receptor expression. Vero cells were cultured in EMEM+5%FBS and PenStrep.

Astrosphere line derived from primary cortical stem cells

Primary radial glia were isolated from developing human cortical tissue. Astrocytes were generated from these cortical stem cells using a previously described *in vitro* method (39, 58). Radial glia were maintained in untreated cell culture flasks with astrocyte media (ASM) (DMEM-F12, 0.5X N2 Supplement, 0.5X B27 -Vit.A Supplement, Heparin [2ng/ml]) plus Y27632 (Tocris) at 10µM. As cells began to form spheroid aggregates, the cultures were maintained in suspension with ASM plus EGF and FGFb (both Peprotech) at 10 ng/ml each and media changed every 4-5 days. Spheroid aggregates were triturated every 7-10 days and transferred to new untreated tissue culture flasks to accommodate proliferation. Around day 160, cultures were validated as astrocyte progenitor cells using immunocytochemistry (ICC) to confirm stellate morphology and canonical astrocyte gene expression. On day 0 of ICC validation, a few spheroids were triturated into a single cell suspension and plated on Matrigel coated glass cover slips with ASM plus CNTF and BMP4 (both Tocris) at 10 ng/ml each. Media was changed every 48 hours. On day 7, cultures were fixed with 4% PFA and processed.

RNA isolation and qPCR

Primary cortical tissue (developing and adult) and organoid samples were collected and processed for RNA extraction using QIAGEN RNeasy Plus Micro Kit. Following the QC, cDNA was prepared using SuperScript™ IV VILO™ Master Mix and qPCR performed for selected genes of interest using LightCycler® 480 SYBR Green I Master Mix. Primer sequences for genes of interest are:

Gene	**Sequence**
GAPDH	F: GGAGCGAGATCCCTCCAAAAT
	R: GGCTGTTGTCATACTTCTCATGG

BSG/CD147	F: GAAGTCGTCAGAACACATCAACG
	R: TTCCGGCGCTTCTCGTAGA
BSG/CD147	F: GGGAGTCAGCGGAGTTCTCCTT
	R: CTAGTAGACTTCTGCACAGACACC
NRP1	F: GCGGCTCACAAAGAATAAGC
	R: ATCCACCAAAACCAACCAAA
DPP4	F: CAAAAACACAGCAAGGGTGA
	R: TAACAGGGCAAGCTGATGTG
DPP4	F: ATGAAGACACCGTGGAAGGTTCT
	R: ACTGTCAGCTGTAGCATCATCTG
TMPRSS2	F: CAAGTGCTCCAACTCTGGGAT
	R: AACACACCGATTCTCGTCCTC
TMPRSS4	F: ATGCGGAACTCAAGTGGGC
	R: CTGTTTGTCGTACTGGATGCT
CTSB	F: ACAACGTGGACATGAGCTACT
	R: TCGGTAAACATAACTCTCTGGGG
FURIN	F: TCGGGGACTATTACCACTTCTG
	R: CCAGCCACTGTACTTGAGGC
LY6E	F: GCCTGAGCAAGACCTGTTCC
	R: CGCACTGAAATTGCACAGAA
IFITM1	F: TCGCCTACTCCGTGAAGTCT
	R: ATGAGGATGCCCAGAATCAG
ACE2	F: TGGAGTTGTGATGGGAGTGA

	R: TCGATGGAGGCATAAGGATT
RNAse P	F: AGA TTT GGA CCT GCG AGC G
	R: GAG CGG CTG TCT CCA CAA GT
SARS-CoV-2 N	F: AAATTTTGGGGACCAGGAAC
	R: TGGCACCTGTGTAGGTCAAC
SARS-CoV-2 E	F: ACAGGTACGTTAATAGTTAATAGCGT
	R: ATATTGCAGCAGTACGCACACA

Table S2: Q-RT-PCR primer sequences for genes of interest utilized in this study.

SARS-CoV-2 infection
The WA-1 strain (BEI resources) of SARS-CoV-2 was used for all experiments and all live virus experiments were performed in a Biosafety Level 3 lab. SARS-CoV-2 stocks were passaged in Vero cells (ATCC) and titer was determined via plaque assay on Vero cells.

SARS-CoV-2 infections at MOI 0.5 were incubated with virus for 2 hours. After inoculation, the media was removed, cells were washed with PBS 2x, culture media was replaced, and cells were incubated at 37°C for 72 hours. Culture media was collected, samples were washed with PBS, and fixed with 4% PFA for 1 hour before removal from the BSL-3 laboratory. MOI was determined by dissociating a parallel organoid or tissue slice, of the same size from each biological sample, and counting the number of cells present in the sample.

For inhibitor experiments, slices were additionally cultured with 100uM Vildagliptin (Sigma, CDS022675), starting 24 hours before infection. The slices were maintained in the media plus inhibitor throughout the span of the experiment.

Plaque assay
Titer of virus isolated post-infection of astrocyte cell line was determined via plaque assay on Vero E6 cell expressing TMPRSS2. Media was collected 72 hours post infection and was diluted 1:10^2-1:10^6 and incubated for 1 hour on Vero cells before an overlay of Avicel and complete DMEM was added. After incubation at 37°C for 72 hours, the overlay was removed and cells were fixed with 10% formalin, stained with crystal violet, and counted for plaque formation.

Immunohistochemistry
Cortical organoids after infection were collected, fixed in 4% PFA for 1 hour, washed with 1xPBS for 2 hours and submerged in 30% sucrose in 1xPBS until saturated. Organoids were embedded in cryomolds containing 50% O.C.T. (Tissue-tek) and 50% of 30% sucrose in 1xPBS and frozen

at -80C. Organoids were sectioned at 10um onto glass slides. Antigen-retrieval was performed on sections using a citrate-based antigen retrieval solution at 100x (Vector Labs) which was boiled, and tissue submerged in solution for 20mins. After antigen retrieval, slides were blocked with PBS containing 5% donkey serum, 2% gelatin and 0.1% Triton X-100 for 1 hour. Primary antibodies were incubated in blocking buffer on slides at 4°C overnight, washed with PBS containing 0.1% Triton X-100 three times and then incubated with AlexaFluor secondary (Thermo Fisher) antibodies at room temperature for 3 hours.

Organotypic slice cultures were fixed for 1 hour in 4% PFA and washed with 1xPBS 2 hours at room temperature. Slices were submerged in 30% sucrose in 1xPBS until saturated and embedded in cryomolds containing 50% O.C.T. (Tissue-tek) and 50% of 30% sucrose in 1xPBS and frozen at -80°C. Slices were sectioned at 10um onto glass slides, subjected to boiling citrate-based antigen retrieval solution (Vector Labs) for 20 min and permeabilized and blocked in blocking buffer (PBS plus 0.1% Triton X-100, 10% donkey serum, and 0.2% gelatin) for 1 h at room temperature. Primary antibodies were diluted in blocking buffer and applied to sections overnight at 4°C. Sections were washed with PBS plus 0.5% Triton X-100 and then incubated in AlexaFluor secondary antibodies (Thermo Fisher and Jackson Labs) diluted in blocking buffer at 4 °C overnight. Images were acquired on a Leica TCS SP5 X laser confocal microscope.

Primary Antibodies include: Mouse: dsRNA, clone rJ2 (Millipore, MABE1134, 1:100), Sox2 (Santa Cruz, sc-365823, 1:500), S100B (Sigma, S2532, 1:200), Ki67 (Abcam, ab156956, 1:500), CD31 (Agilent, GA61061-2, 1:100), Olig2 (Millipore, MABN50, 1:100), Ctsb (Santa Cruz, sc-365558, 1:100), Gapdh (Calbiochem, CB1001, 1:3000), RFP (Thermofisher, MA5-15257, 1:300), Rabbit: SARS-CoV-2 (Sino Biological, 40143-R001, 1:200), Pax6 (Biolegend, 901301, 1:500), Hopx (Proteintech, 11419-1-AP, 1:500), cleaved-Caspase-3 (Cell Signaling, 9661S, 1:250), Synm (Proteintech, 20735-1-AP, 1:100), Aqp4 (Proteintech, 16473-1-AP, 1:600), Egfr (Abcam, ab32077, 1:100), Dpp4 (Proteintech, 10940-1-AP, 1:50), CD147 (Invitrogen, 34-5600, 1:500), Furin (Novus Biologicals, NB100-1903, 1:300), Ttr (Agilent Daki, A000202-2, 1:100), Arcn1 (Proteintech, 23843-1-AP, 1:50), GLAST (Proteintech, 20785-1-AP, 1:200), Ifitm3 (Thermo Fisher, PA5-11274, 1:200), CD14 (abcam, ab183322, 1:500), RFP (Invitrogen, R10367, 1:500), Rat: Gfap (Thermofisher, 13-0300, 1:200), Laminin (Abcam, ab80580, 1:500), Nrp1 (Abcam, ab81321, 1:50), Chicken: Gfap (Abcam, ab4674, 1:500), Map2 (Abcam, ab5392, 1:200), Trem2 (Millipore, MABN755, 1:100) Goat: Ace2 (R&D, AF933, 1:200), Ace2 (Thermofisher, MA5-32307, 1:200), Iba1 (Abcam, ab48004, 1:500), Pdgfrb (R&D, AF385, 1:100), Sheep: Eomes (R&D, AF6166, 1:200), Guinea pig: NeuN (Millipore, ABN90, 1:500), Iba1 (Synaptic Systems, 234 004, 1:500) Sheep: CD34 (R&D, AF7227, 1:200).

Bulk RNA sequencing and differential expression analysis

RNA was extracted from a GW21 and GW23 primary cortical sample after 3 days of infection, as before. RNA was sequenced through Genewiz using 150bp paired end RNA-seq on a Illumina HiSeq platform. Reads were aligned and quantified using kallisto and collapsed to gene level quantifications with tximport (59, 60). Differential expression analysis was performed using limma/voom, accounting for 2 individuals with blocked replicates within each individual. P values were corrected for multiple comparisons using the Benjamini-Hochberg false-discovery rate

adjustment (60, 61) (SI Appendix Table 1).

Pathway and gene set enrichment analysis

Four gene sets were used for pathway analysis. For SARS-CoV-2 infection versus control, we defined a set of upregulated genes (FDR < 10%, 100% increase in expression, 178 genes) and downregulated genes (FDR < 10%, 50% reduction in expression, 23 genes). For SARS-CoV-2 dosage sensitive genes, we used only the samples with SARS-CoV-2 infection and correlated the expression level of the SARS-CoV-2 E and N genes (as calculated by qPCR) with gene expression, and identified correlated (R > 0.8) and anti-correlated (R < -0.8) gene sets. We took the intersection of genes for the E and N genes as the final set of dose-responsive genes. Gene Ontology pathway term enrichment was performed using TopGO (62). Cell-states were identified using data from the supplementary tables in Refs (24, 25). The LPS-stimulated activated microglia set was defined from Table S2 in Drager et al., using genes with fold change > 2 and FDR < 5%. The additional cell states were defined using the cluster-specific genes from the CROP-seq experiment delineated in Table S5 from that study. The IL-alpha/TNF/C1q stimulated reactive astrocyte set was defined from Table S1 in Leng et al., with fold change > 2 and FDR < 5%. The reactive astrocyte states were defined using the cluster-specific genes from CROP-seq in Table S8 from that study.

TUNEL assay

Slides were post-fixed in 4% PFA in TBS for 20 mins at room temperature then washed for 30 mins in TBS, before being permeabilized for 2 mins with 0.1% Triton X-100. Slides were washed 2X for 3 mins each in TBS. TUNEL mix from the In Situ Cell Death Detection Kit - Fitc (Roche) composed of TUNEL label and TUNEL enzyme were mixed in a proportion of 10:1 and added to slides for 1 hour at 37C. Slides were washed 3X in TBS at room temperature for 5 mins each before being coverslipped as before.

In situ hybridization

Primary fixed lung and cortical tissue samples were processed using the protocol for RNAscope Multiplex Fluorescence Assay v2 (Advanced Cell Diagnostics Catalog# 323100) for human ACE2 amplification, targeting nucleotides 307 – 1267 of NM_021804.3 (Advanced Cell Diagnostics Catalog 848151-C2) or SARS-CoV-2 Spike, targeting nucleotides 21631 – 23303 of NC_045512.2 (Advanced Cell Diagnostics Catalog 848561-C3). For human CD4 amplification, targeting nucleotides 1726 - 2734 (Advanced Cell Diagnostics Catalog 605601). Antibody post-staining was performed as before.

Western blotting

Protein lysates were extracted from a range of tissues and cell lines, in RIPA buffer (Thermo Fisher, 89900) and complete protease inhibitor cocktail (Millipore-sigma, P8340-1ML). Normal human astrocytes were cultured either with 10% FBS or with FBS and BMP4, CNTF(63). Vero kidney epithelial cells were used as positive control, along with primary lung tissue. Protein lysates in Lämmli-buffer containing 2-mercaptoethanol, were boiled for 10′. Protein samples were separated using 12% SDS-poly-acrylamide gels and transferred to Nitrocellulose membranes (Protan, GE). Primary antibodies α-ACE2 (R&D), α-DPP4 (Proteintech), α-CD147 (Invitrogen)

and α-GAPDH (Calbiochem) were incubated with the membrane overnight at 4°C. Secondary antibody horseradish peroxidase conjugated α-Goat-Ig and α-Mouse-Ig (Jackson Immunoresearch Labs) incubation was performed for 1.5h at RT. Detection was done by chemiluminescence.

Lentiviral knockdown
shRNAs directed against receptors ACE2, DPP4 and CD147 were cloned in pLKO-RFP vector backbone and lentiviruses generated and packaged in 293T cells. Supernatant was collected after 48 and 72hours. Lentiviral particles were concentrated by ultracentrifugation. Primary astrocytes were infected with these lentiviruses, kept in culture for four days and the efficiency of knockdown tested by RT-qPCR.

shACE2_1	CCGGGCTGGACAGAAACTGTTCAATCTCGAGATTGAACAGTTTCTGTCCAGCTTTTTG
shACE2_2	CCGGGCCCTTATTTACCTGGCTGAACTCGAGTTCAGCCAGGTAAATAAGGGCTTTTTG
shDPP4_1	CCGGGCCCAATTTAACGACACAGAACTCGAGTTCTGTGTCGTTAAATTGGGCTTTTTG
shDPP4_2	CCGGCCAGAAGACAACCTTGACCATCTCGAGATGGTCAAGGTTGTCTTCTGGTTTTTG
shCD147_1	CCGGCCAGAATGACAAAGGCAAGAACTCGAGTTCTTGCCTTTGTCATTCTGGTTTTT
shControl	pLKO-RFP (Addgene, plasmid 69040, sequence- CAACAAGATGAAGAGCACCAA)

Table S3: Sequences for shRNA constructs used to make lentiviruses.

Overexpression Plasmids
cDNAs for DPP4 and CD147 amplified from pLEX307-DPP4-puro and BSG-bio-His plasmids, were subcloned in the corrected pCDH-EF1a-mCherry lentiviral vector backbone. Lentiviruses were generated and packaged in 293T cells. The overexpression efficiency was tested in organoid samples post lentiviral infection by q-RT-PCR. pCDH-EF1a-eFFly-mCherry was a gift from Irmela Jeremias (Addgene plasmid # 104833 ; http://n2t.net/addgene:104833 ; RRID:Addgene_104833,(64)). pLEX307-DPP4-puro was a gift from Alejandro Chavez & Sho Iketani (Addgene plasmid # 158451 ; http://n2t.net/addgene:158451 ; RRID:Addgene_158451). BSG-bio-His was a gift from Gavin Wright (Addgene plasmid # 53146 ; http://n2t.net/addgene:53146 ; RRID:Addgene_53146, (65)).

Quantification strategy
All infected (SARS-CoV-2 N+, dsRNA+, SARS-CoV-2 S+) cells within a section were imaged in combination with cell marker genes. Proportions of cell types infected by SARS-CoV-2 were counted using ImageJ software. The number of infected cells positive for a given marker (ex GFAP), as indicated by co-localization of either SAR-CoV-2 N or dsRNA, were counted (ex GFAP+dsRNA+). The number of double positive cells per image were calculated over the total

number of infected cells (ex GFAP+dsRNA+/total dsRNA+). Each data point represents a stained tissue sample, where data was collected and averaged across at least three fields of view per image. Quantification for changes in reactivity and stress markers were counted both from mock and MOI 0.5 infected samples. The number of cells positive for each reactivity marker were calculated over the total number of Dapi+ cells. Inhibitor experiments were quantified by evaluating the total number of SARS-CoV-2 N+ or dsRNA+ cells within the MOI 0.5 and MOI 0.5+Vildagliptin samples.

Supplemental Figures

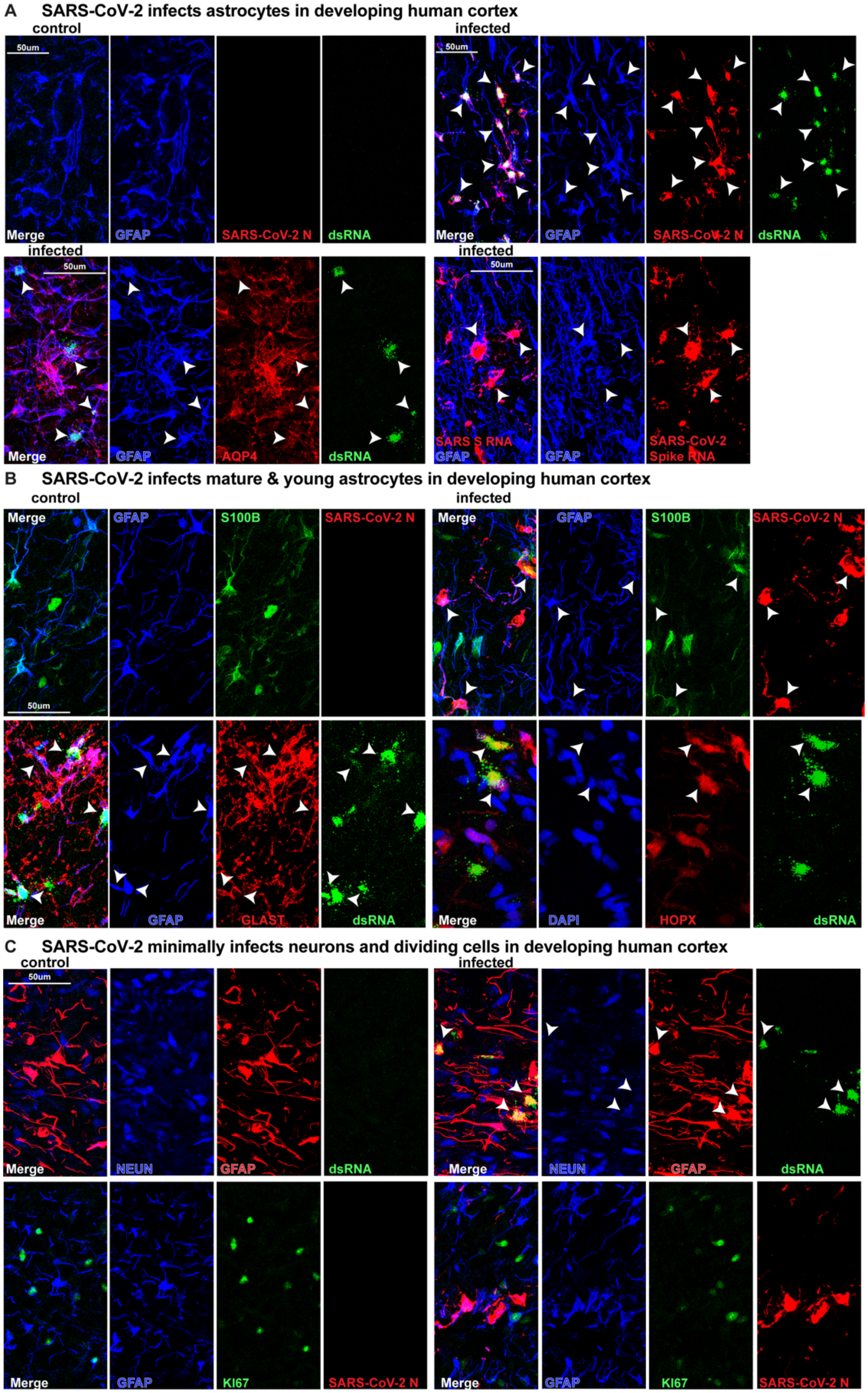

S Figure 1. SARS-CoV-2 infects astrocytes in developing human cortex

A) Single and merge channel images from Figure 1B demonstrating co-expression of SARS-CoV-2 infection and astrocyte markers. **B)** Split channel images from Figure 1D indicating co-expression of infection and astrocyte markers GLAST, S100B, and HOPX. **C)** Split channel images from Figure 1E indicating SARS-CoV-2 minimally infects NEUN+ neurons and KI67+ dividing cells.

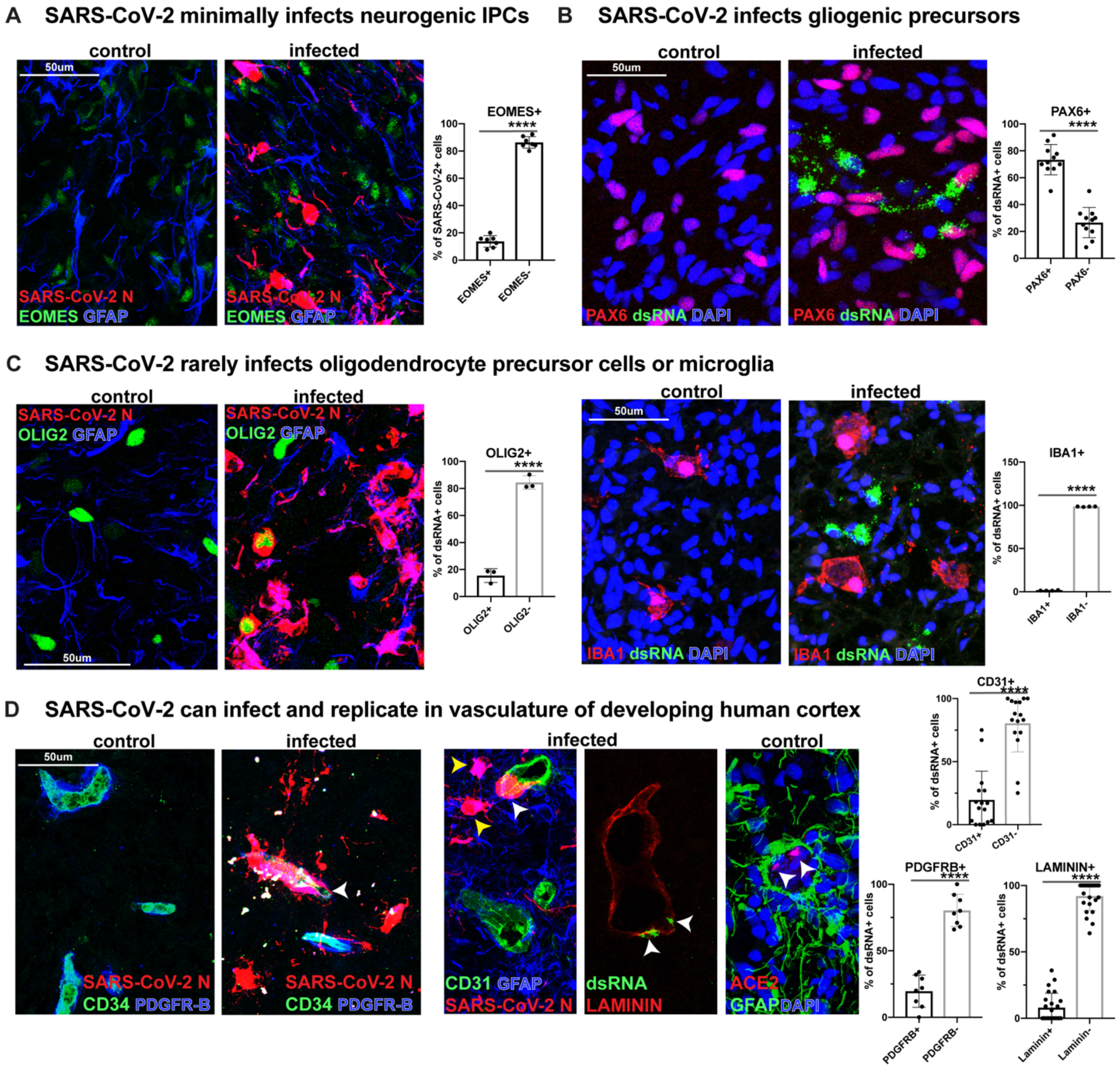

S Figure 2. Non-astrocyte neural and vascular cell types have minimal tropism for SARS-CoV-2

A) In organotypic slice cultures after 2 hours of SARS-CoV-2 exposure and 72 hours of culture, neurogenic intermediate progenitor cells (IPCs) show minimal infection where less than 14% of infected cells are IPCs (EOMES: unpaired student's t-test: ****p<0.0001, n=3 biological samples across 6 technical replicates). **B)** PAX6+ astroglial precursors are readily infected by the SARS-CoV-2 virus where 74% of infected cells express PAX6 (PAX6: unpaired student's t-test: ****p<0.001, n=3 biological samples from 6 technical replicates). **C)** Other glial cell types, like OLIG2+ Oligodendrocyte Precursor Cells (OPC) and IBA1+ microglia, are minimally infected compared to astrocytes. About 15% of SARS-CoV-2+ dsRNA+ infected cells are OLIG2+ and about 1% are IBA1+ (OLIG2: unpaired student's t-test: ****p<0.0001, n=3, IBA1: unpaired student's t-test: ****p<0.0001, n=4 technical replicates from 2 biological samples). **D)** Vascular cells including CD31+ endothelial cells, PDGFRB+ mural cells, and LAMININ+ blood vessels can

be infected by SARS-CoV-2 (white arrowheads). Vascular cell types indicate a lower infection rate, of about 20%, compared to astrocytes (Laminin: unpaired unpaired student's t-test: ****p<0.0001 CD31: ****p<0.0001, PDGFRB: ****p<0.0001 n=3 biological samples and >6 technical replicates, error bars represent SD). GFAP+ astrocytes surround vascular cells which can express the SARS-CoV-2 entry factor, ACE2. Astrocytes located adjacent to vasculature are also infected (yellow arrowhead).

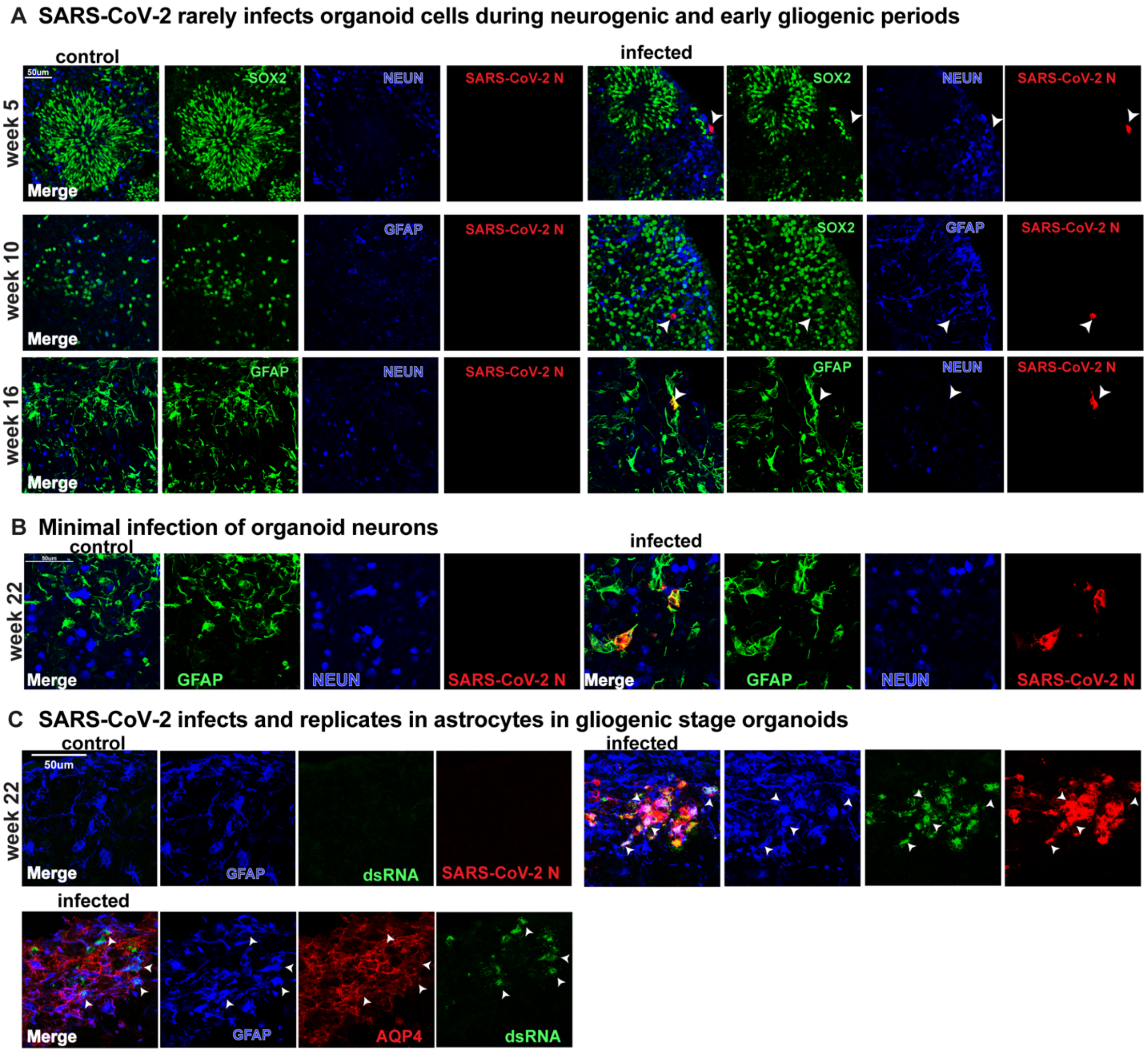

S Figure 3. SARS-CoV-2 infects astrocytes in cortical organoids

A) Merge and single channel images from Figure 2B demonstrating co-expression of SARS-CoV-2 infection during neurogenic and gliogenic periods. **B)** Single channel images from Figure 2D indicating co-expression of SARS-CoV-2 infection and astrocyte marker, GFAP, but no infection in NEUN positive neurons. **C)** Merge and single channel images from Figure 2E indicating SARS-CoV-2 infection and replication in astrocytes in gliogenic stage organoids.

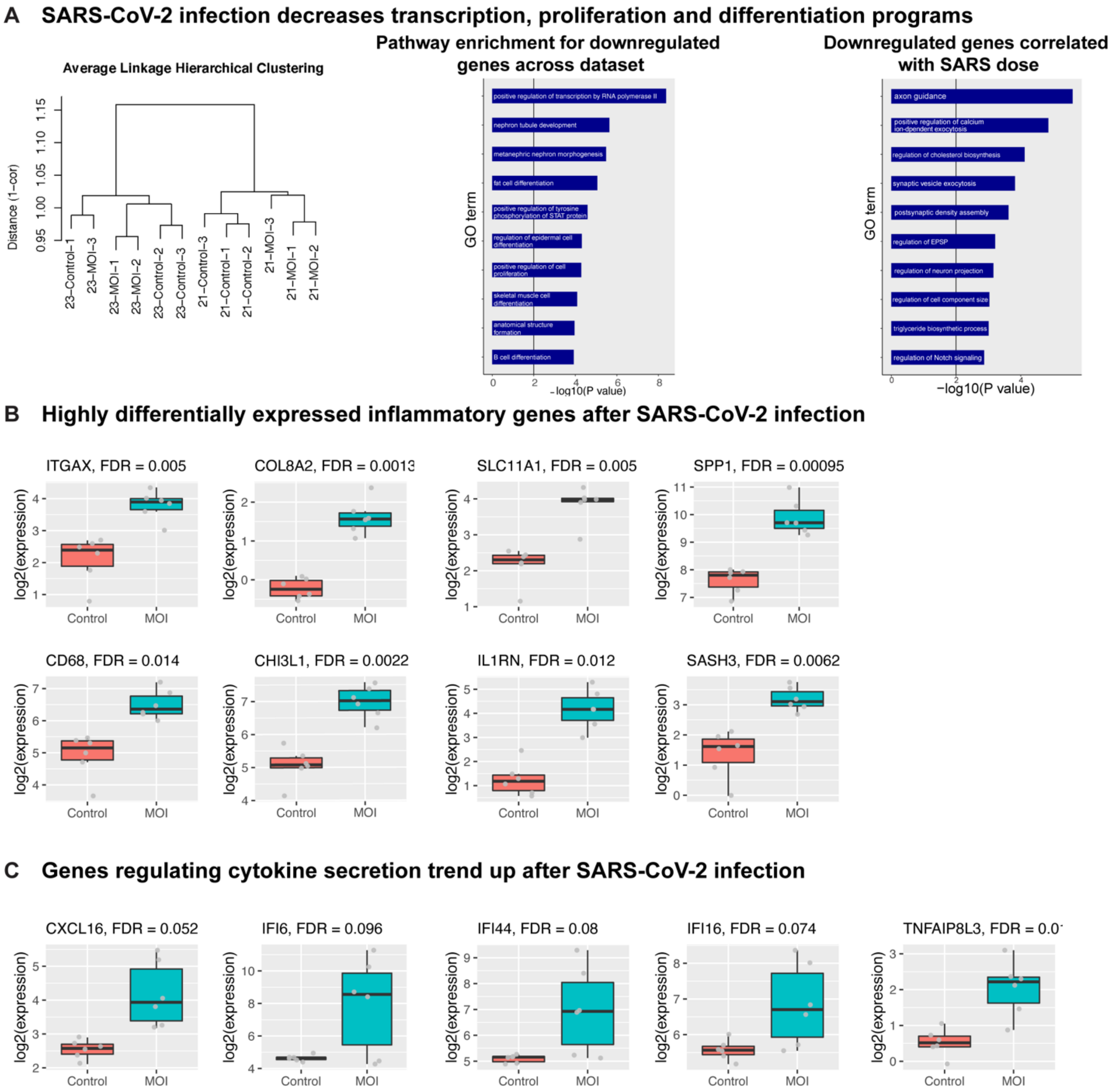

S Figure 4. RNA sequencing reveals increased inflammatory response and cytokine signaling after SARS-CoV-2 infection

A) Bulk RNA sequencing dataset from primary infected organotypic cultures. Left: Average-linkage hierarchical clustering of samples using (1-[Pearson Correlation]) as the distance. Distance is first driven by individual/age and then infection. Middle: Pathway enrichment analysis using gene ontology. Downregulated set of 172 genes based on FDR < 0.1 and at least a 2 fold decrease (50% of normal expression). Downregulated genes regulate transcription and cellular development. Right: Downregulated set of 836 genes based on FDR < 0.1 and at least a 2 fold decrease (50% of normal expression) correlated with SARS-CoV-2 dose. Downregulated genes correspond to neural development programs. **B)** Box plots with individual expression values for differentially expressed genes regulating inflammation and microglia activation: ITGAX

(FDR<0.005) COL82 (FDR<0.0013), SCL11A1 (FDR<0.0005), SPP1 (FDR<0.00095), CD68 (FDR<0.014), CHI3L1 (FDR<0.0022), IL1RN (FDR<0.012), SASH3 (FDR<0.0062). **C)** Box plots with individual expression values for differentially expressed genes involved in cytokine secretion: CXCL16 (FDR<0.052), IFI6 (FDR<0.096), TNFAIP8L3 (FDR<0.01), IFI44 (FDR<0.08), IFI16 (FDR<0.074).

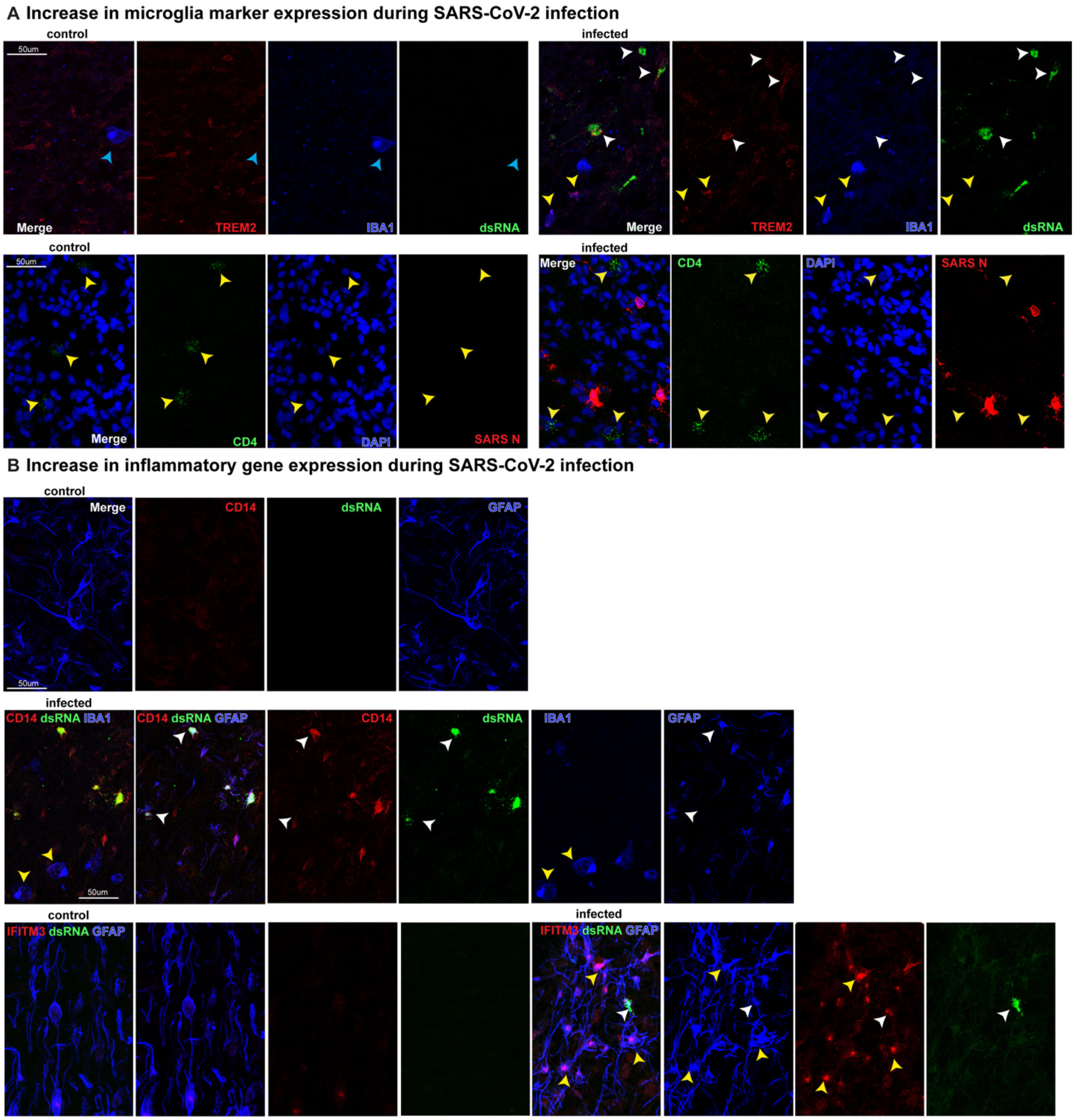

S Figure 5. RNA sequencing reveals an inflammatory response in human cortical tissue after SARS-CoV-2 infection

A) Merge and single channel images from Figure 3C demonstrating increase in microglia markers, TREM2 and CD4, after SARS-CoV-2 infection. **B)** Split channel images for Figure 3D demonstrating increase in inflammation markers, CD14 and IFITM3, post-SARS-CoV-2 infection.

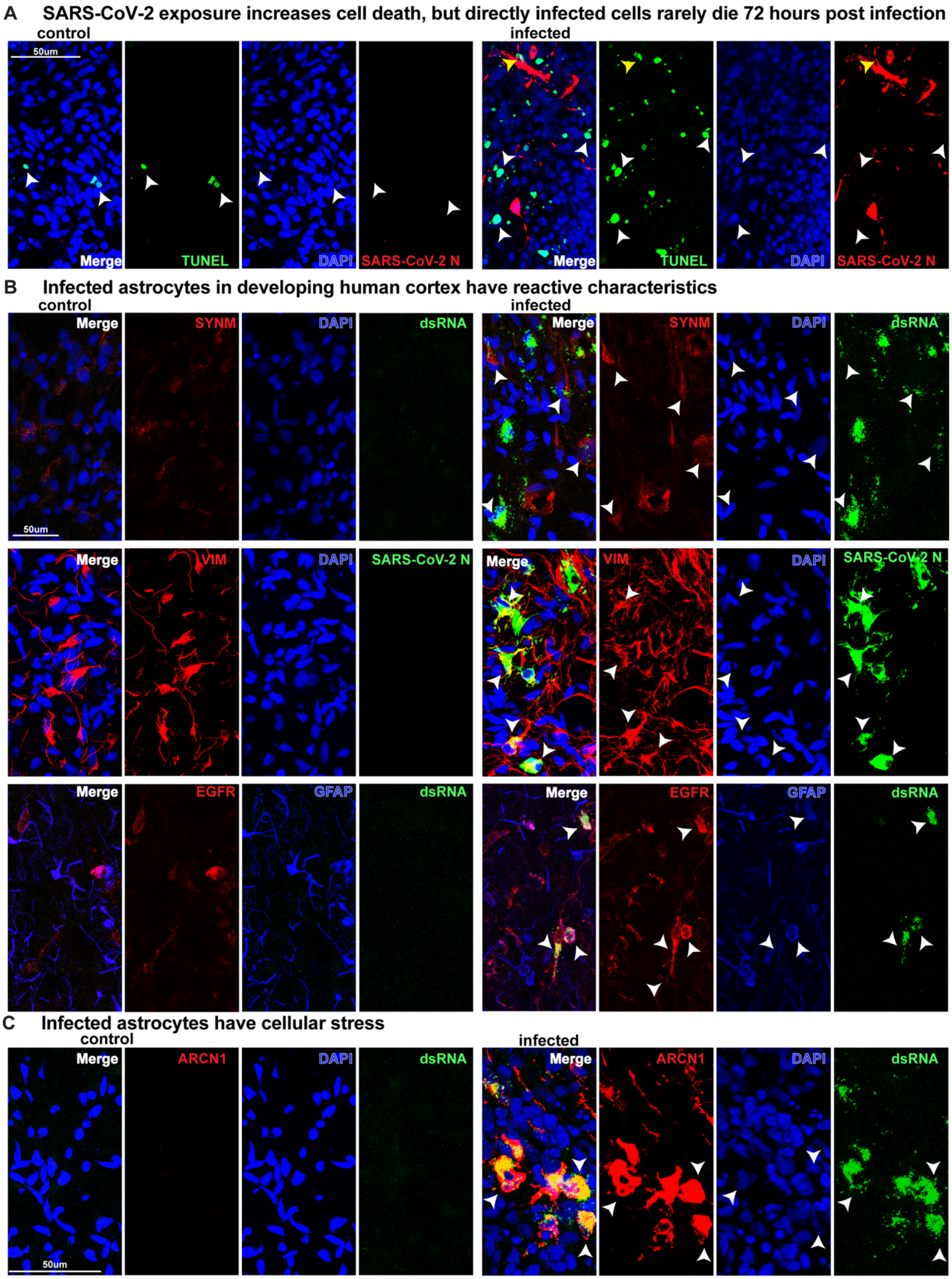

S Figure 6. SARS-CoV-2 infection increases cell stress and reactivity in cortical astrocytes

A) Merge and single channel images from Figure 4A demonstrating increase in TUNEL+ dying

cells in organotypic slice cultures after SARS-CoV-2 infection. **B)** Split channel images from Figure 4B demonstrating increase in reactivity in cortical astrocytes, tested with markers SYNM, VIM, and EGFR. **C)** Single channel images from Figure 4C demonstrating cell stress, indicated by ARCN1 expression, in astrocytes post-SARS-CoV-2 infection.

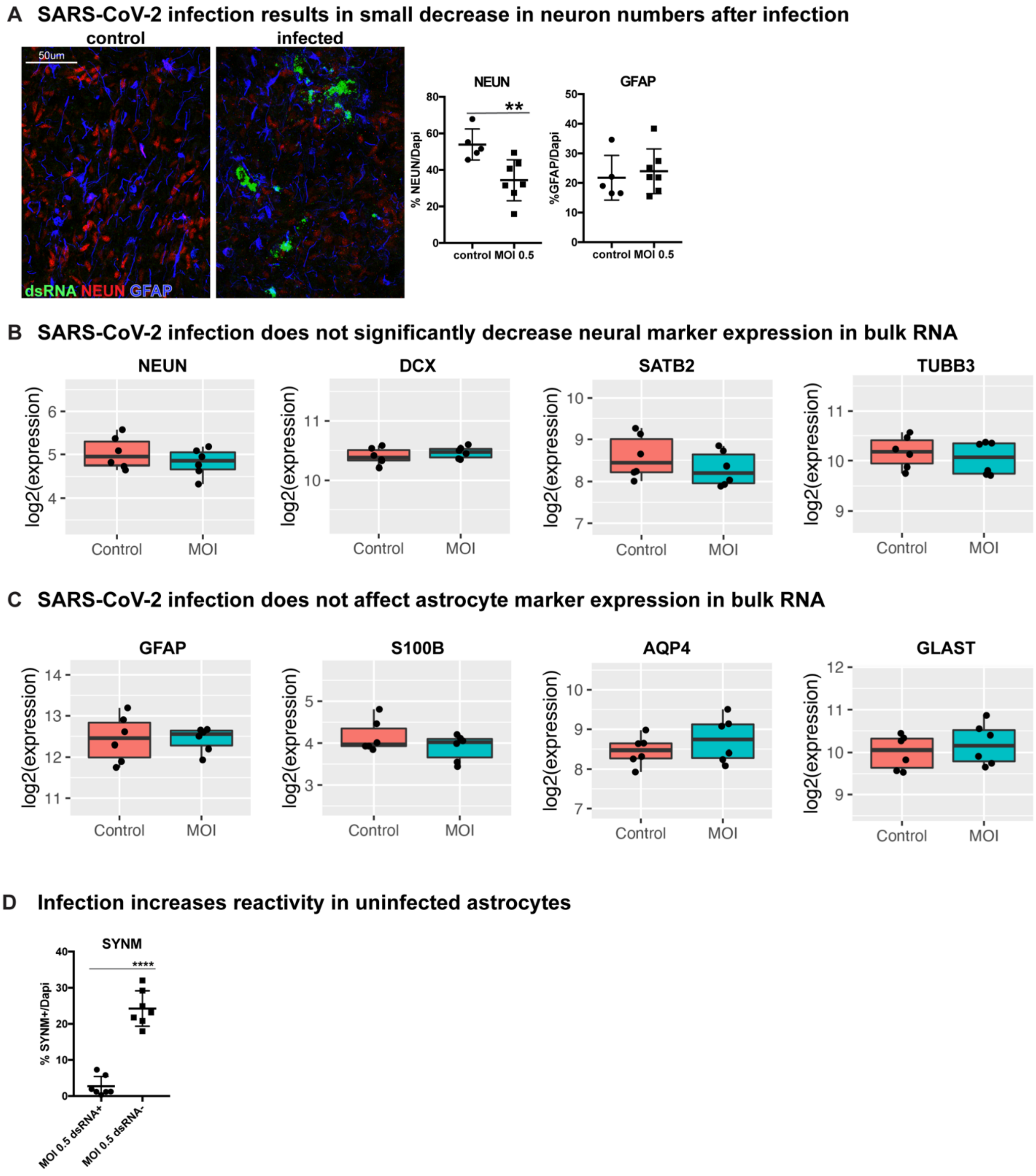

S Figure 7. SARS-CoV-2 infection has cell non-autonomous impact on neurons and astrocytes

A) In primary slice cultures exposed to SARS-CoV-2, three days post-infection there is a small decrease in the number of NEUN+ neurons. There are no significant change in the number of GFAP+ astrocytes after three days post-infection (Unpaired student's t-test, NEUN: **$p<0.0084$, GFAP: $p=0.632$, n=2 biological replicates and across 3 technical replicates). **B)** Bulk RNA sequencing of slice cultures infected by SARS-CoV-2 do not indicate significant differences

between control and infected groups for expression of neuronal markers: NEUN, DCX, SATB2 or TUBB3. **C)** Bulk RNA sequencing does not indicate significant differences between control and infected groups for expression of astrocyte markers: GFAP, S100B, AQP4, GLAST. **D)** A marker of astrocyte reactivity, SYNM, increases in surrounding un-infected astrocytes (Unpaired student's t-test, ****p<0.0001, n=2 biological replicates and across 3 technical replicates).

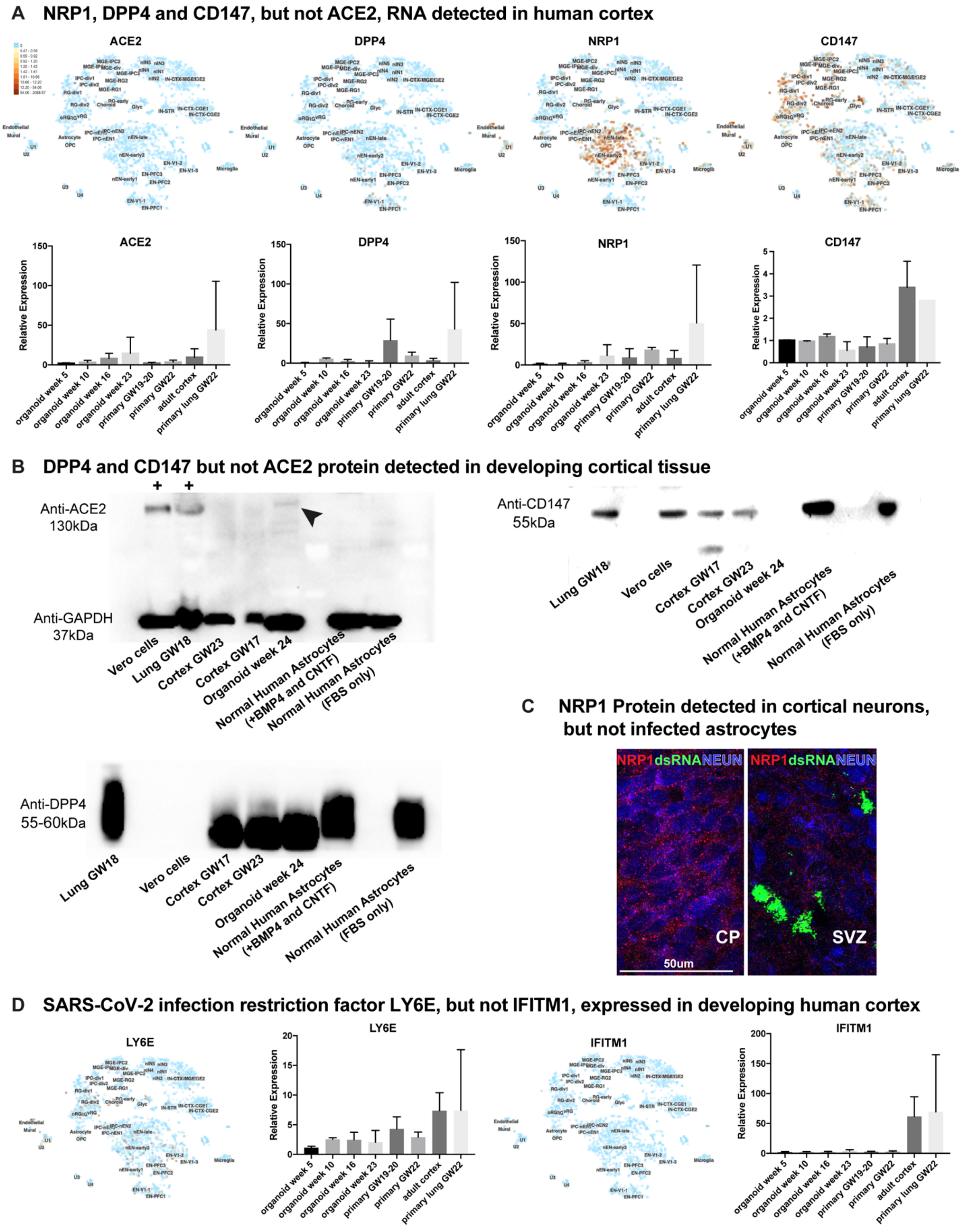

S Figure 8. Coronavirus infection cofactors are expressed in developing human cortex

A) Re-analysis of single cell RNA sequencing data from Nowakowski et al., 2017 and bulk RNA

expression of ACE2, DPP4, NRP1 and CD147 across stages of cortical organoids, developing cortex, adult cortex and developing lung suggest there is minimal ACE2 RNA in the human brain. DPP4, NRP1, and CD147 are expressed in neural sample types (n>2 biological sample/sample type, n>2 technical replicates per sample/gene). Gapdh was used to calculate relative expression of each gene of interest. **B)** Western blot of ACE2, DPP4 and CD147. Vero cells and developing lung (positive controls) indicate high ACE2 abundance. Cortical tissue and normal human astrocytes (NHA), indicated no ACE2 protein. There may be a small quantity of ectopic ACE2 in organoids (arrowhead). CD147 and DPP4 are robustly abundant in human neural tissue and cells (n=2 technical replicates/sample/blot). **C)** NRP1 is present in cortical neurons in the cortical plate, but is not observed in infected cells in the SVZ. **D)** Restriction factors necessary for coronavirus infection are also expressed in the developing cortex, where LY6E has broad expression and IFITM1 is only expressed in adult stages (n>2 biological sample/sample type, n>2 technical replicates per sample/gene).

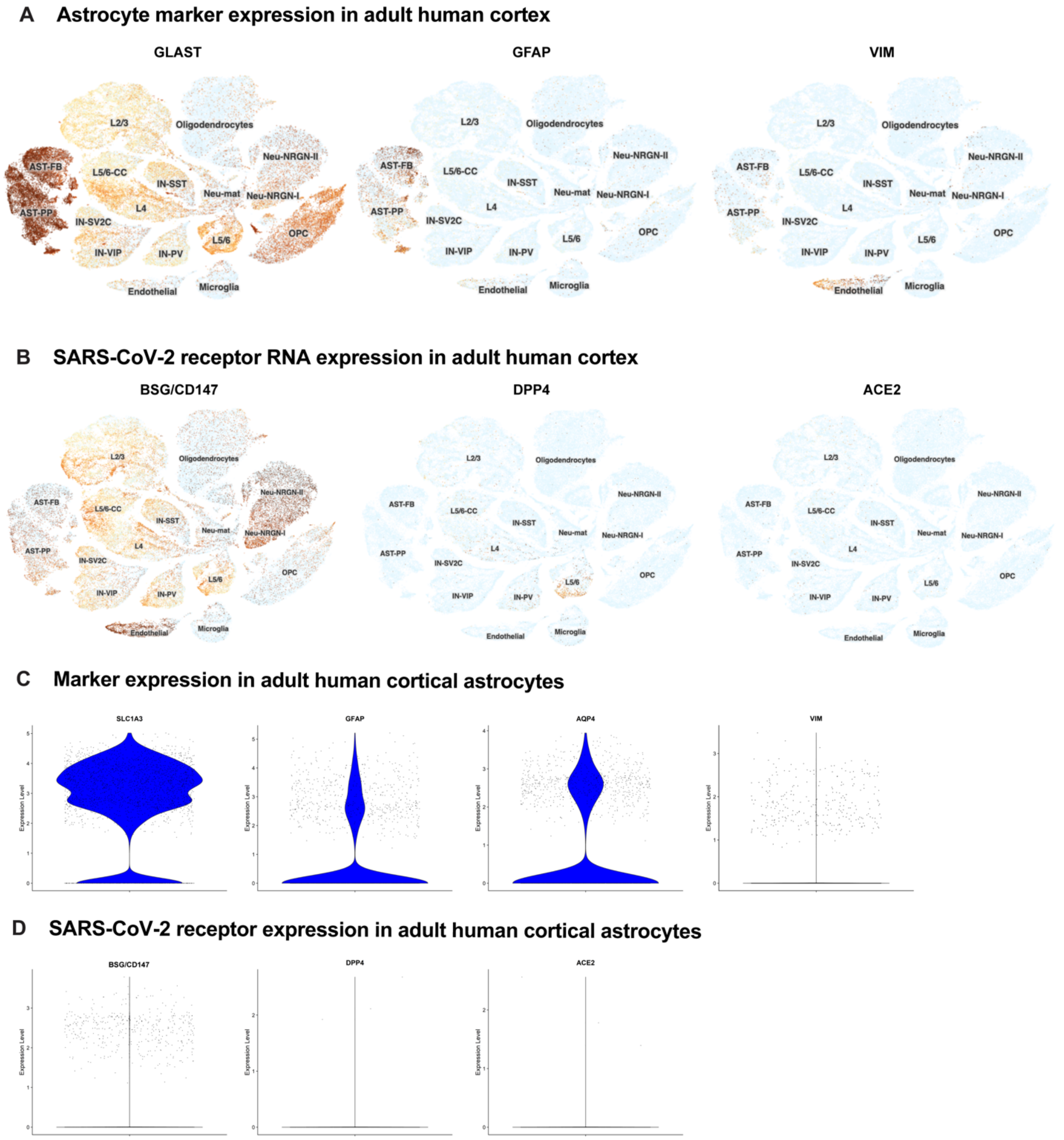

S Figure 9. Adult astrocyte marker gene expression and SARS-CoV-2 expression

A) Single Cell RNA sequencing data of human cortical samples collected from 4 - 22 years of age (55) indicating GLAST, GFAP, and VIM expression. **B)** Abundance of BSG/CD147, DPP4, and ACE2 in the same dataset. **C)** Violin plots of relative expression of SLC1A3 (GLAST), GFAP, AQP4 and VIM in cortical astrocytes from the (55) dataset. **D)** There is modest RNA expression of BSG, but minimal DPP4 or ACE2 in adult astrocytes.

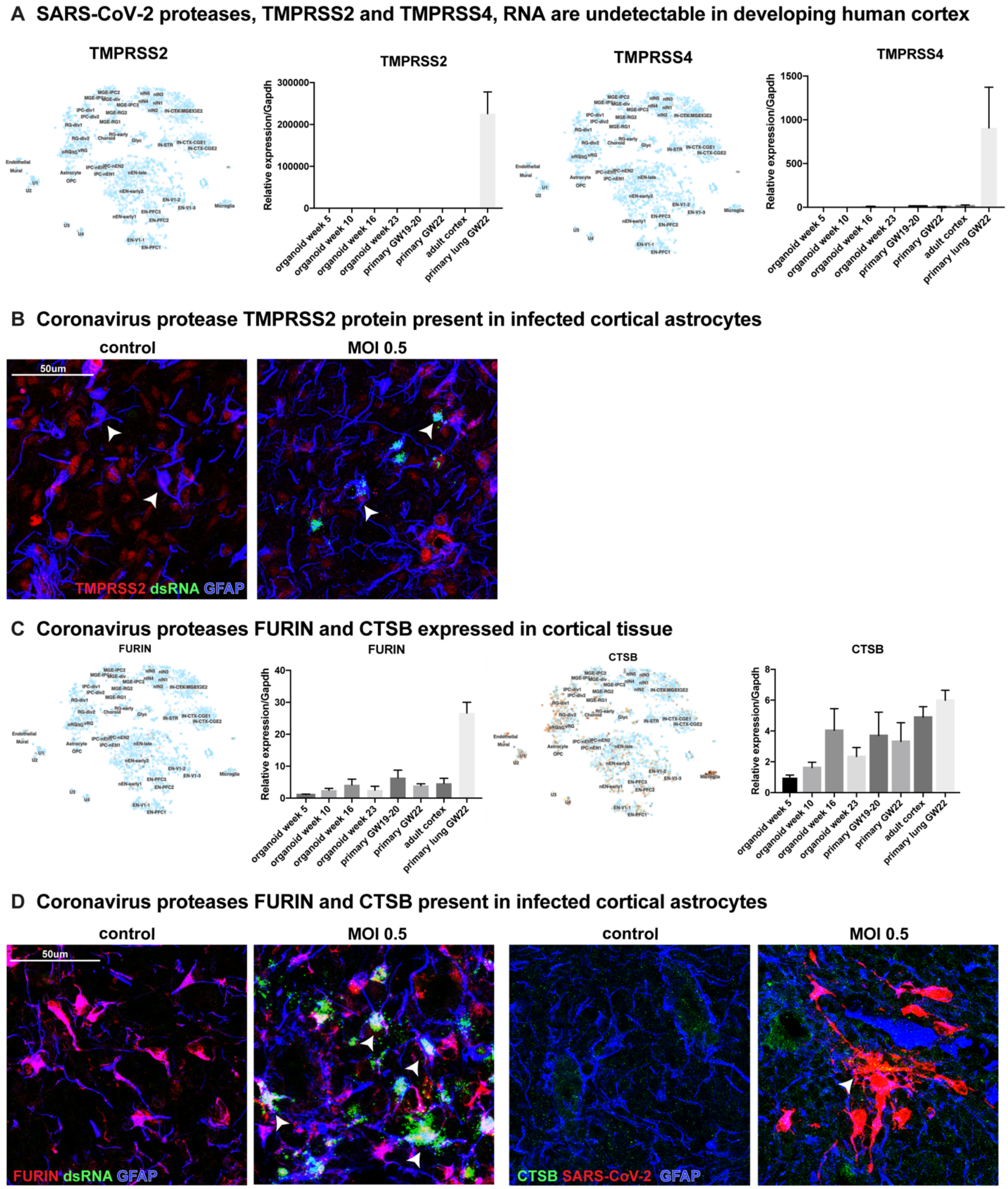

S Figure 10. Coronavirus protease expression in developing human cortex

A) Single-cell RNA sequencing data and qRT-PCR for bulk RNA from developing cortical samples demonstrates minimal expression of canonical SARS-CoV-2 proteases, TMPRSS2 and TMPRSS4, in cortical cells compared to developing lung (n>2 biological sample/sample type, n>2 technical replicates per sample/gene). **B)** Despite low RNA abundance, TMPRSS2 protein is

present in cortical astrocytes and other cortical cell types**.** White arrowheads indicate TMPRSS2+ astrocytes in control and infected samples (n=2 biological samples). **C)** Alternative coronavirus proteases, FURIN and CTSB, are differentially expressed in the developing human cortex (n>2 biological sample/sample type, n>2 technical replicates per sample/gene). **D)** FURIN and CTSB protein are both present in developing cortical tissue. White arrowheads indicate FURIN+ or CTSB+ infected astrocytes (n=2 biological samples).

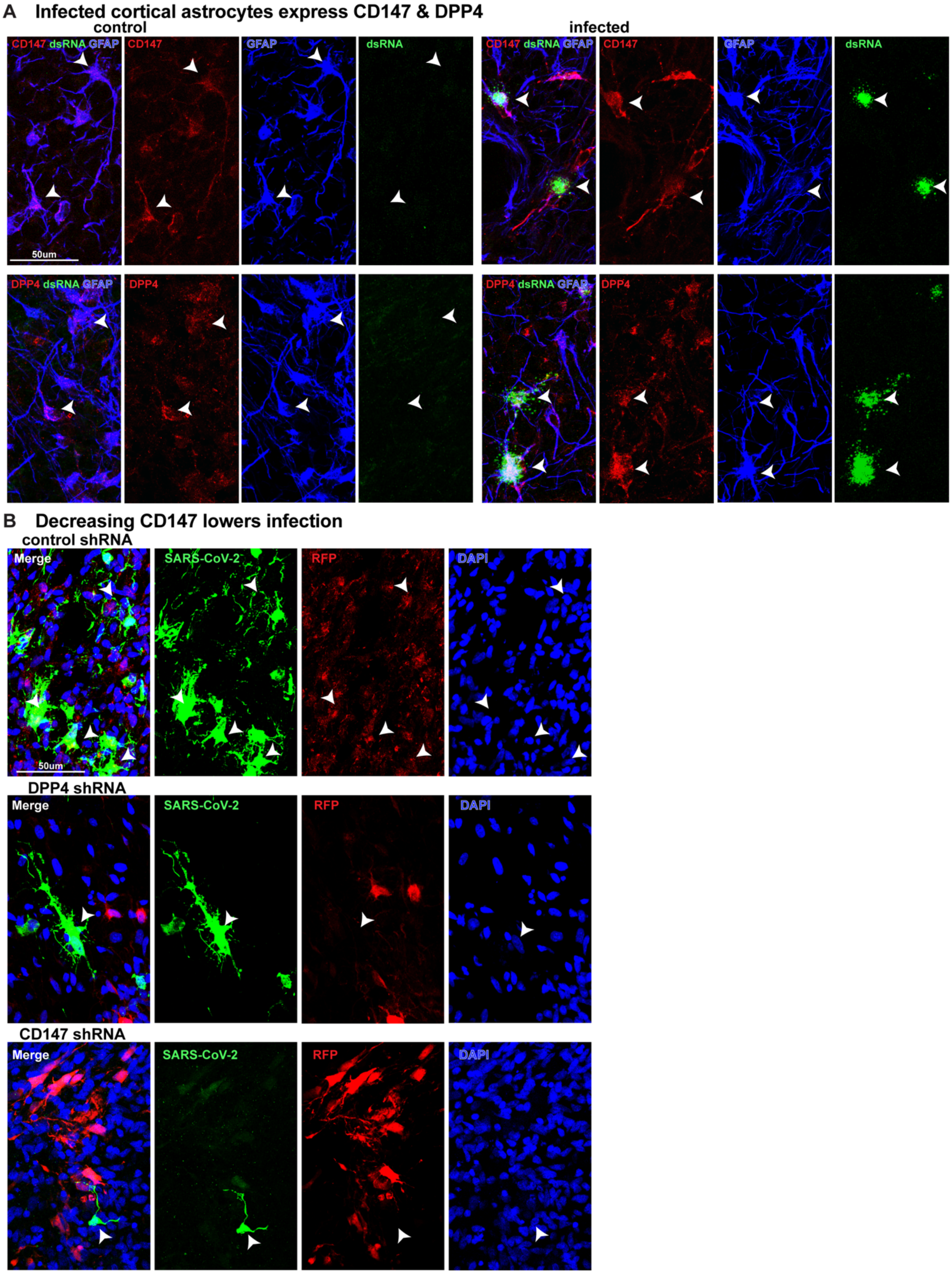

S Figure 11. Coronavirus receptors, CD147 and DPP4, but not ACE2 are expressed in developing human cortex

A) Merge and single channel images of CD147 and DPP4 expression on cortical astrocytes from Figure 5C. **B)** Merge and single channel images from Figure 5D indicate that knocking down CD147 and DPP4 expression impacts infection rate.

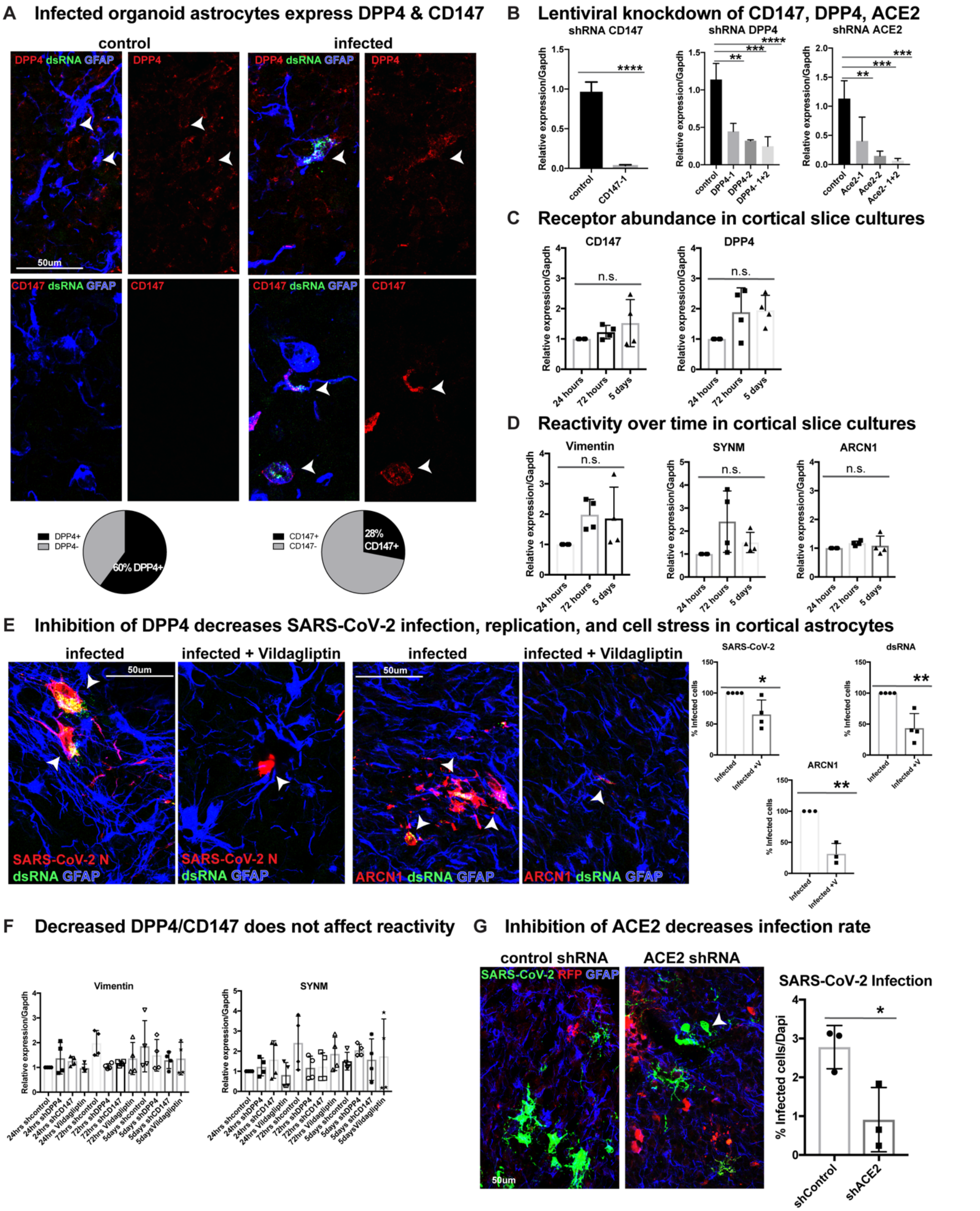

S Figure 12. Coronavirus receptors are present in organoids and astrocyte cell lines and are responsive to pharmacological manipulation

A) In cortical organoids, 60% of dsRNA+ infected cells are DPP4+ and 28% are CD147+ (n=2 cell lines across 4 technical replicates **B)** Lentiviral constructs containing shRNAs directed against

ACE2, DPP4 and CD147 were cloned and packaged. Lentiviral knockdown was validated in primary astrocytes (CD147 n=6, DPP4 n=6, ACE2 n=4 samples from two independent experiments; Unpaired student's t-test: control vs CD147-1 ****p<0.00001; One-way ANOVA: control vs DPP4-1 **p<0.0012, control vs DPP4-2 ***p<0.0003, control vs DPP4-1+2 ****p<0.0001, control vs ACE2-1 **p<0.0042, control vs ACE2-2 ***p<0.0003, control vs ACE2-1+2 ***p<0.0001). **C)** Expression of DPP4 and CD147 in cortical slice cultures over time (One-way ANOVA, DPP4: 24hr vs 72 hours p=0.11, 24 vs 5 days p=0.084; CD147: 24hr vs 72 hours p=0.78, 24 vs 5 days p=0.30, error bars indicate SD, n=4). **D)** Reactivity markers do not significantly increase over time in cortical slice cultures (One-way ANOVA, VIM: p=0.133, SYNM: p=0.095, ARCN: p=0.499, error bars indicate SD, n=4). **E)** Inhibition of DPP4 by Vildagliptin results in a decrease of SARS-CoV-2 N+ and dsRNA+ cells (white arrowheads, student's t-test: SARS-CoV-2: *p<0.0255, dsRNA: **p<0.003, error bars represent SD n=2 biological samples from 4 technical replicates). ARCN1 is reduced after DPP4 inhibition (white arrowheads, student's t-test: **p<0.002, MOI 0.5 vs MOI 0.5+ Vildagliptin n=2 biological samples and 3 technical replicates). **F)** Knockdown of DPP4 and CD147 by shRNAs or inhibition by Vildagliptin does not affect expression of VIM or SYNM (n=4). **G)** Lentiviral knockdown of ACE2 decreases SARS-CoV-2 infection rate compared to control shRNA (Unpaired student's t-test: ACE2 *p<0.032, n=2 independent experiments).

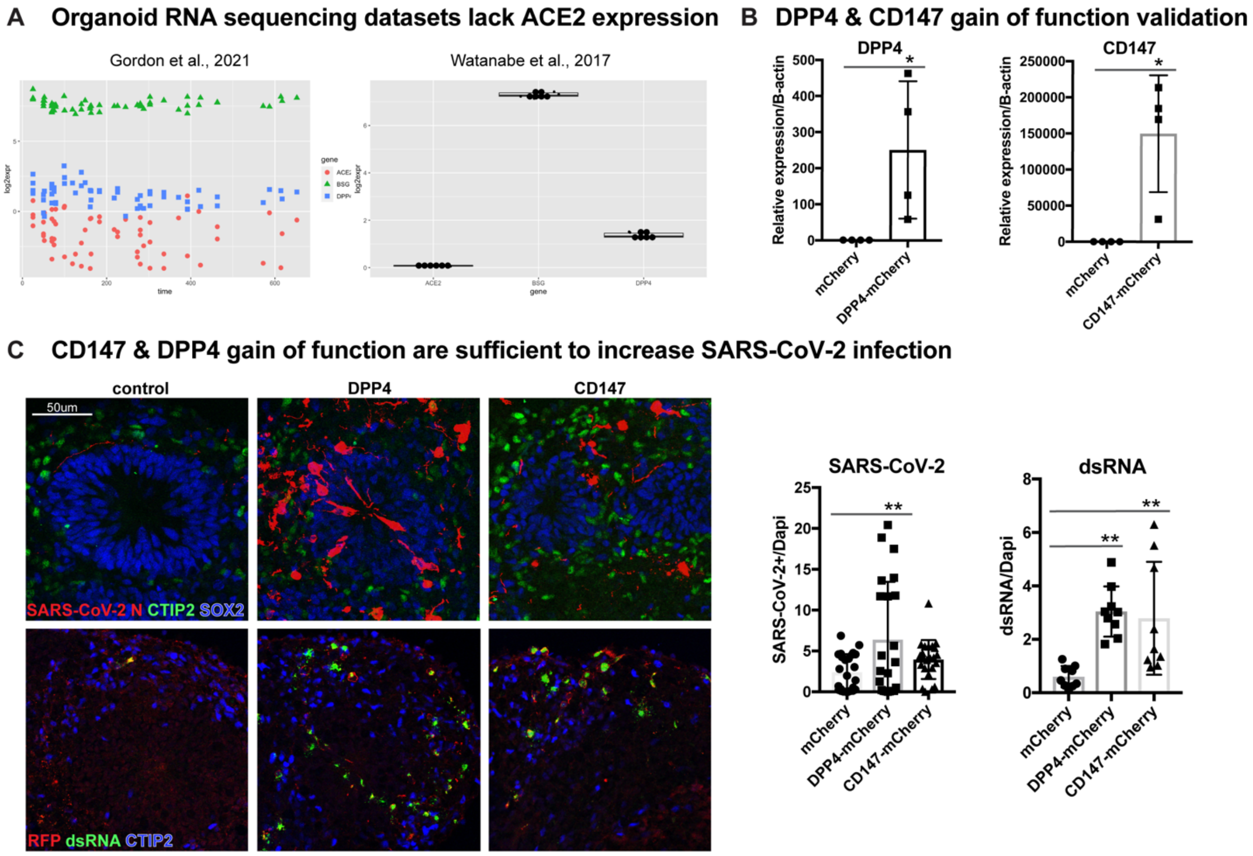

S Figure 13. Sufficiency of DPP4 and CD147 to mediate infection

A) Publicly available organoid RNA sequencing datasets lack ACE2 (56, 57). **B)** DPP4 and CD147 plasmids were subcloned and packaged in lentiviruses. Week 5 organoids were infected and a significant increase of expression was observed (unpaired student's t-test: DPP4: *$p<0.0393$, CD147: *$p<0.0101$, n=4 technical replicates from 2 lines across 2 independent experiments, error bars represent SD). **C)** After an increase in DPP4 or CD147 expression there was an increase in SARS-CoV-2 N and dsRNA (One-way ANOVA: SARS-CoV-2 mCherry vs DPP4 **$p<0.0075$; dsRNA mCherry vs DPP4 **$p<0.0023$, mCherry vs CD147 **$p<0.0061$, n=3 organoids/line from 2 lines).

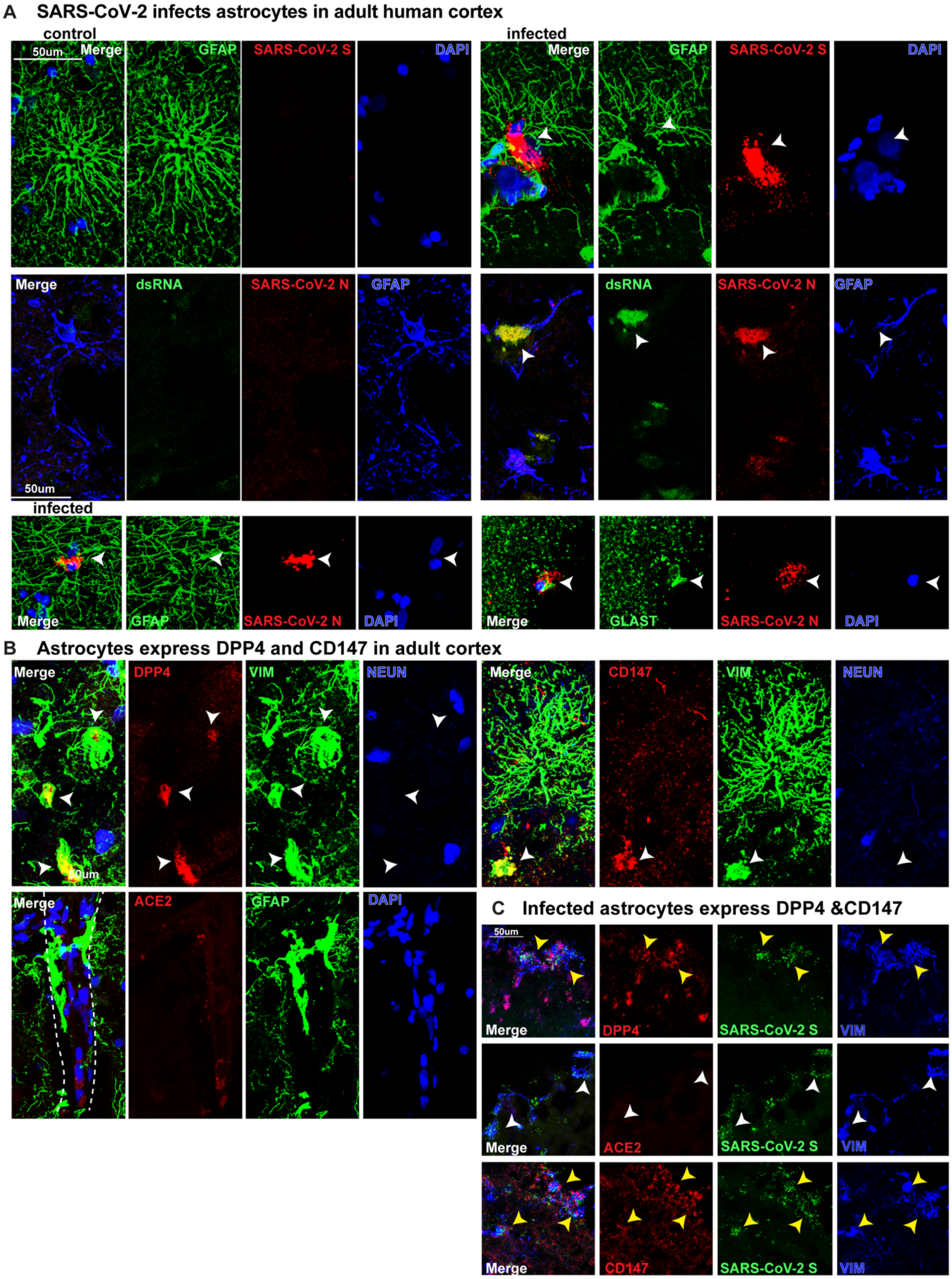

S Figure 14. Astrocytes in the adult human cortex are vulnerable to SARS-CoV-2 infection

A) Merge and single channel images from Figure 6A indicating infection in GFAP+ GLAST+

astrocytes. **B)** Split channel images from Figure 6B demonstrate CD147 and DPP4 expression in adult VIM+ astrocytes. **C)** Split channel images from Figure 6C indicating expression of DPP4 and C147, but not ACE2 on infected VIM+ astrocytes.
